# Optimizing water and nitrogen management improves maize productivity by regulating root development in the cold semi-arid Songnen plains of Northeast China

**DOI:** 10.3389/fpls.2025.1658353

**Published:** 2025-09-19

**Authors:** Yu Xin Chi, Ihsan Muhammad, Shahid Ali, Mohammad Shah Jahan, Li Yang, Xun Bo Zhou

**Affiliations:** ^1^ Guangxi Key Laboratory of Agro-environment and Agro-products Safety, Guangxi Colleges and Universities Key Laboratory of Crop Cultivation and Tillage, Agricultural College, Guangxi University, Nanning, China; ^2^ Guangxi Key Laboratory of Forest Ecology and Conservation, State Key Laboratory for Conservation and Utilization of Agro-bioresources, College of Forestry, Guangxi University, Nanning, Guangxi, China

**Keywords:** maize, enzymes, crop yield, nitrogen, root development, water stress

## Abstract

**Introduction:**

Water constraints and excessive nitrogen (N) application hinder root development in spring maize in cold semi-arid regions (CSR), limiting growth and yield. In this study, we focused on the CSR where water scarcity and high fertilizer use are major challenges. Optimizing water-N interactions can enhance root distribution and nutrient uptake, offering a key strategy for improving crop productivity.

**Materials and methods:**

To determine the optimal water-N management strategy under local climatic conditions and evaluate its effects on root physiology and yield performance of spring maize. A two-year field experiment (2020-2021) tested four N application rates (N0: 0 kg ha^-1^, N100: 100 kg ha^-1^, N200: 200kg ha^-1^, N300: 300 kg ha^-1^) and three soil moisture levels (S1: 40%, S2: 60%, S3: 80% field capacity). Water was managed by maintaining target soil moisture using TDR-based measurements and supplemental irrigation.

**Results:**

Compared with S3-N300, S3-N200 increased plant height (7.89%), stalk thickness (10.48%), and spike position height (5.14%), while substantially boosting root antioxidant enzymes (7.72%), lowering reactive oxygen species (11.81%) and raising K^+^ (18.22%), Ca^2+^ (16.35%), Mg^2+^ (20.01%), and reduced Na^+^ (3.83%) levels. It also elevated Indole-3-acetic acid (IAA), Gibberellins (GAs), and Zeatin + Zeatin Riboside (Z+ZR) by 45%, 43%, and 30%, respectively. Biomass accumulation rose in spike (11.98%), leaf (23.21%), stalk (16.63%), and grain (6.95%), resulting in 8.01% yield improvement. Structural equation modeling (SEM) showed that water-N interactions explained 94% of the variation in yield, 89% in ion content, 94% in hormones, and 91% in ROS levels.

**Conclusion:**

These findings confirm that S3-N200 (80% field capacity + 200 kg N ha^-1^) treatment improved root function, stress resilience, and nutrient uptake, thereby enhancing growth and yield compared to conventional local practice (>250-300 Kg N ha^-1^) without optimized water management. Optimizing water-N strategies in North China’s CSR supports sustainable maize production and strengthens agricultural resilience under water-limited conditions.

## Graphical Abstract

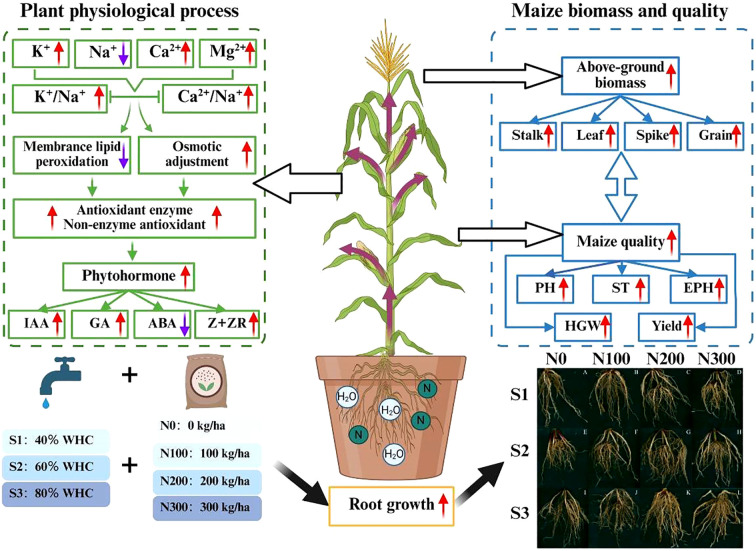

## 1 Introduction

Maize (*Zea mays* L.) is one of the primary food sources for a significant portion of the world’s population (FAO, 2020). Globally, maize is ranked as the third most important cereal crop after wheat and rice. Global maize consumption continues to rise, with enhancing pressure to maintain high yield under resource-limited and climate-stressed conditions (Erenstein et al., 2022). In cold semi-arid regions (CSR) of northern China, maize production is constrained by low precipitation, high evapotranspiration, and limited water availability, by imbalanced fertilization use. Water stress from both inadequate irrigation or drought is a dominant environmental factor that restricts crop growth, development, and reduces crop yield (Muhammad et al., 2025). Excessive N fertilization, mostly used to compensate for low soil fertility, which can further deteriorate soil health, increase N leaching, lodging risk, and contribute to environmental pollution (Muhammad et al., 2022a). Therefore, proper water-N management strategy is crucial for improving resource use efficiency and enhancing sustainable maize production under CSR environmental conditions. In this scenario, root development plays a crucial role in plant adaptation to water and nutrient availability during vegetative growth, especially in arid and semi-arid regions (Araus et al., 2008). The nutrients acquired by roots are essential for maize plant growth, and a restricted nutrient supply can hinder plant growth, leading to discernible phenotypical changes (Hu and Chu, 2020).

Water stress is one of the most critical environmental factors regulating plant growth and development and limiting crop production in CSR. Additionally, plants can respond and adapt to water stress by altering their cellular metabolism and activating various defense systems (Muhammad et al., 2022b). Plant survival under stress conditions relies on perceiving stimuli, generating and transmitting signals, and initiating various physiological and chemical changes (Wang et al., 2016, Wang et al., 2018). The mitotic activity of plant cells was always inhibited under drought stress. The drought-induced inhibition of maize leaf growth is associated with a significant reduction in the number of cells in the meristematic tissue (Sun et al., 2023). Meanwhile, drought stress also causes excessive accumulation of ROS in plants, which causes secondary oxidative stress, membrane damage, electrolyte leakage, lipid peroxidation, and consequently plant cell death (Voothuluru et al., 2020). Drought stress reduces N accumulation in roots, thereby restricting root growth and their distribution (Xia et al., 2021a). Although total root biomass reduces during drought stress, however, it increases the proportion of roots in deep soil layers, which favors water and nutrient absorption and stable grain yields (Xia et al., 2021b). Excessive irrigation adversely affects maize growth during waterlogging, where soil oxygen deficiency hampers root growth and premature plant aging, which ultimately reduces grain yield (Wang et al., 2019).

Nitrogen limitation hinders the growth and productivity of crops, resulting in the expansion of root architecture, an increase in biomass, and changes in root exudate composition (Zhang W, et al., 2021). Likewise, N deficiency triggers root branching and elongation to improve soil exploration, while high N fertilization alters root metabolic processes and morphology (Schlüter et al., 2012). These studies primarily focus on probing particular classes of metabolites, such as amino acids or fatty acids. However, capturing the aggregate effect of maize root physiology on biomass production under both N-deficient and sufficient conditions is essential for understanding metabolism throughout the plant. Plants have evolved inherent mechanisms to regulate growth and development in response to various environmental stresses. The control of cell expansion plays a crucial role in water stress responses and plant growth (Maggio et al., 2006). Additionally, cell growth caused by cell expansion is regulated by turgor pressure, which is the physical force against the cell wall and is maintained by osmotic regulation through osmotically active substances, including potassium ions (K^+^), magnesium ions (Mg^2+^), calcium ions (Ca^2+^), sugars, and amino acids (Xia et al., 2013). During water deficit stress, osmotic stress sensing and signaling are essential for maintaining plant water status, leading to rapid changes in gene expression (Yamaguchi-Shinozaki and Shinozaki, 2006; Osakabe et al., 2011) and turgor-dependent stomatal closure, which responds to hydraulic properties in the xylem (Aksu and Altay, 2020; Roelfsema et al., 2012).

To understand plant adaptation to N availability, systems biology methodologies are essential to comprehensively study the metabolic response of maize roots under N-deficient and N-sufficient conditions. Root systems have evolved structural and physiological strategies to forage resources in complex soil environments (Lynch, 2015; York et al., 2013). Efficient use of soil water and nutrients significantly influences crop growth and yield, which largely depends on root morphological traits and their temporal and spatial distributions (Ortega, 2010). Understanding root phenotypes under variable N regimes, contrasting water conditions, and their interactions, is crucial for integrating root phenotypes in crop breeding (Lynch, 2019). However, the impact of optimizing combined N and water management on maize yield through enhancing the spatial match between roots and soil water distribution remains unclear.

The purposes of this study were: (1) to examine the interactive effect water and N on root distribution, growth dynamics, morphology, osmotic adjustment, oxidative stress, and maize yield; (2) to identify the most effective water-N interaction for cold and semi-arid areas of north China; (3) to optimize N application levels to enhance water use efficiency and support sustainable maize production. We hypothesized that the interaction of optimized water and N levels would enhance root development and physiological function, thereby improving nutrient uptake, stress tolerance, and ultimately increasing maize yield in cold semi-arid regions.

## 2 Materials and methods

### 2.1 Experimental site, plant materials, and growing conditions

**Figure 1:**
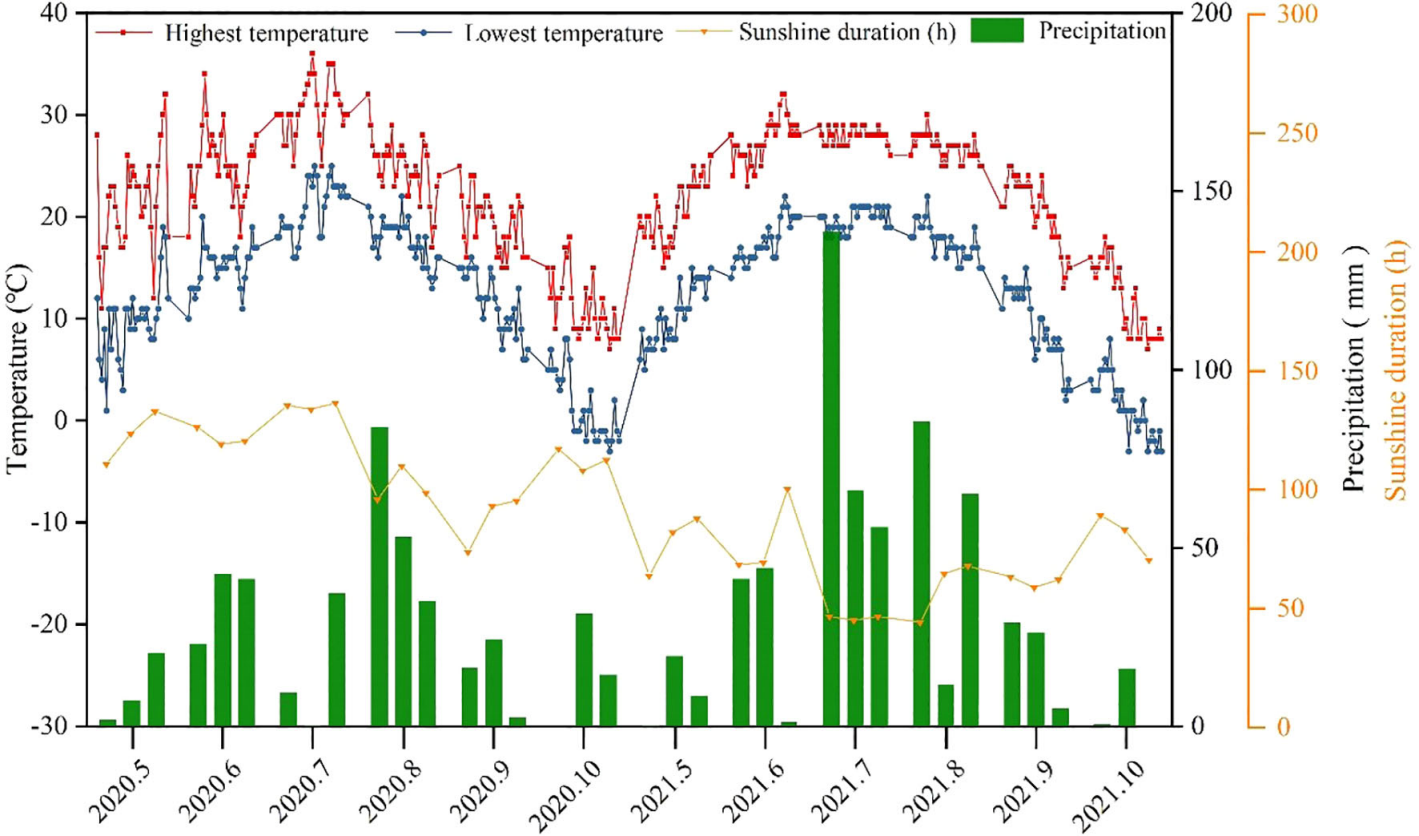
Temperature and precipitation of the upper, middle, and lower ten days per month and daily sunshine duration in 2020-2021.

This study was conducted at the experimental station of the Qiqihar Branch, Heilongjiang Academy of Agricultural Sciences, China (47°16′26″N, 123°41′46″E), located in a semi-arid region of the western Songnen plain. The soil was classified as Aeolian sandy, with a depth of 0–20 cm, a pH of 7.82, a bulk density of 1.26 g cm^2^, and an organic matter content of 26.52 g kg^-1^. Available N, phosphorus (P), and potassium (K) were 100.05, 16.91, and 134.03 mg kg^-1^, respectively. **Figure 1** shows the daily temperature, precipitation, and sunshine duration during the maize growing and fallow seasons of 2020 and 2021. The mean annual air highest temperature, lowest temperature, sunshine duration, and precipitation of the experimental site were 22.4°C, 12.7°C, 664.1 h, and 604 mm, respectively.

*Zea mays L.*, hybrid cultivar Nendan 19 (ND19) was used as a test crop. Plants were established at a density of 75,000 plants ha^-1^, with row to row spacing of 65 cm and plant to plant spacing of 25 cm, consistent with field conditions. To ensure experimental accuracy, soil samples were collected from conventional farms and subjected to a drought-resistant environment. Intact and uniform seeds (germination rate > 90%) were selected for planting. Each pot, measuring 29.5 cm in diameter and 38 cm in height, was filled with 30 kg Aeolian sandy soil collected from normal farmland, and the planting arrangement replicated field spacing.

### 2.2 Experimental design

The experiment consisted of four N rates: 0 kg N ha^-1^ (N0), 100 kg N ha^-1^ (N100), 200 kg N ha^-1^ (N200), and 300 kg N ha^-1^ (N300), and three water treatment levels: S1 (40% of field holding capacity (FHC), S2 (60% FHC), S3 (80% FHC). This factorial design resulted in twelve distinct treatments, each replicated thrice, comprising thirty-six experimental units. Fertilization included urea (46.4% N) applied at 0, 1.02, 2.02, and 3.02 g pot^-1^ corresponding to N0: 0 kg ha^-1^, N100: 100 kg ha^-1^, N200: 200kg ha^-1^, N300: 300 kg ha^-1^; P_2_O_5_ at100 kg ha^-1^ (18% P at 3.59 g pot^-1^); and KCl at100 kg ha^-1^ (60% K at 3.59 g pot^-1^). Two-thirds of the fertilizer was applied as base fertilizer at sowing, and the remaining one-third was applied at the 12th leaf stage. Soil moisture was measured using a TDR-100 meter (Spectrum Technologies Inc., California, USA), with readings taken daily at 06:00 and 18:00. If water conditions do not meet test requirements, artificial irrigation is required. Soil moisture was measured in the 0–20 cm layer. Before rainfall events, the experiment utilized automatically operated rain shelters with a triple-folding mechanism to shield the test area from natural precipitation. Insecticides were deemed unnecessary, but herbicides were applied before and after the planting. Seeds were manually sown on May 12, and harvesting occurred on September 25 during the 2020 and 2021 growing seasons.

## 3 Measurements and calculations

### 3.1 Plant sampling and data collection

The whole maize plant was harvested and washed with distilled water at the maturity stage. Plant height was recorded from the base of the maize stem to the highest point with a ruler, and stem diameter was measured with a Vernier Caliper. The plant were dried at 105°C for 30 min and then dried to a constant weight at 80°C for 72 h for dry weight calculation.

### 3.2 Root length, surface area, tips, diameter, and root-to-shoot ratio measurements

The roots were scanned using the LA-S plant root fine analyzer (MRS-9600TFU2L). LA-S Plant Root Analysis System was then used to analyze the data. The root length density (RLD) and root surface area density (RSD) were calculated using the following equations (Wu et al., 2022).


$$\text{RLD}=\frac{\text{Root lengh}}{\text{Soil volume}}$$

$$\text{RSD}=\frac{\text{Root surface area}}{\text{Soil volume}}$$

$$\text{Root to shoot ratio}=\frac{\text{Root dry weight}}{\text{Shoot dry weight}}$$

### 3.3 Determination of metal ions

To determine the Na^+^, K^+^, Ca^2+,^and Mg^2+^ concentrations in the roots, approximately 0.1 g of fresh root samples were ground in liquid N. Inductively coupled plasma mass spectrometry was used to determined Na^+^, K^+^, Ca^2+,^and Mg^2+^ concentrations in roots (mg kg^-1^) (ICP-MS, NexION 350x, PerkinElme Co., America).

### 3.4 Hormone content

Different treatments were applied to root samples for measuring hormone contents, including GA, IAA, ABA, and Z+ZR. Approximately 0.5 g of maize roots was ground in a mortar with 5 mL of methanol extraction buffer and 1 mmol L^-1^ dibutyl hydroxytoluene as an antioxidant. The extract was allowed to stand at 4°C for 4 h and then centrifuged at 10,000 × g for 15 minutes at 4°C. The supernatant was passed through a Chromosep C18 column, pre-washed with 10 mL of 100% methanol, and then washed with 5 mL of 80% methanol. The extracted hormones were dried in 1 mL of phosphate buffer and then dissolved in 0.1% (v/v) N_2_ between 20% (surfactant) and 0.1% (w/v) gelatin (pH 7.5) for enzyme analysis and immunoassay evaluation (ELISA).

### 3.5 Assay of osmoregulation substance, ROS, and antioxidant activity

Soluble sugar (SS), proline (pro), soluble protein (SP), superoxide anion (O_2_^-^), malondialdehyde (MDA), and H_2_O_2_ content were determined using the method of Muhammad et al. (2023).

The antioxidant enzyme activities of superoxide dismutase (SOD), peroxidase (POD), catalase (CAT), ascorbate peroxidase (APX), glutathione peroxidase (GPX), and glutathione reductase (GR) were detected according to the method by Ahmad et al. (2022).

To determine antioxidant enzyme activities, 0.2 g of fresh leaf sample was homogenized with 5 mL of phosphate buffer (0.1 M, pH 6.8). The mixed liquid sample was centrifuged at 12,000 × g for 20 min at 4 °C. The resultant supernatants were used for SOD, POD, CAT, APX, GPX, and GR activities, and the results were presented as U mg^−1^ min^−1^ FW.

### 3.6 Grain yield

At the physiological maturity stage (R6), all of the remaining ears were harvested to determine yield (length of the spike, spike diameter, rows per ear, and grain per row were counted manually from the selected cobs after the measurement of 100-grain weight, and moisture content was approximately 14%).

### 3.7 Statistical analysis

The experimental data were sorted and calculated using Excel 2019 (Microsoft Corp., Redmond, WA, USA). They were subjected to an analysis of variance (ANOVA) using SPSS 21.0 Statistics (Ver. 21.0, SPSS Inc., Chicago, IL, USA). Comparisons between means were conducted using the Least Significant Difference (LSD) test at *p* < 0.001 and *p* ≤ 0.05. OriginLab 2021 (Northampton, MA, USA) was used to illustrate the figures.

Spearman correlation analysis was used to determine the relationships among environmental variables, maize root growth indexes, and yields, using the R package “Mantel”, and the correlation results were visualized by the ggplot2, dplyr, corrplot, RColorBrewer, and ggsci mixed function of the R package “linKET”. AMOS 24.0 was used to construct structural equation models (SEMs) and test the relationship between different variables (including regression analysis, factor analysis, correlation analysis, and variance analysis). The optimal SEMs with statistical significance were selected for path construction.

## 4 Results

### 4.1 Interactive effects of nitrogen and water on plant growth performance

Significant effects of water treatments, N fertilization, and their interaction were observed for plant height (PH), stalk thickness (ST), and spike position height (SPH) (*p* < 0.05; **Table 1**). Similar trends were recorded in both years (2020 and 2021)., Water stress notably reduced PH, ST, and EPH under S1 and S2 conditions compared with S3 (**Table 1**). The N200 treatment increased PH, ST, and SPH compared with N100 by 7.08%, 3.69%, and 1.93% under S1; 11.79%, 9.62%, and 4.86% under S2; 15.25%, 25.39%, and 9.95% under S3, respectively. Conversely, N300 treatment decreased PH, ST, and EPH by 7.39%, 4.70%, and 1.57%, respectively (**Table 1**). While N variability and water treatments significantly affected PH (*p* < 0.05), no significant interaction was observed between year and N levels (*p >* 0.05). However, the water-N interaction was highly significant (*p* < 0.01), indicating that the availability of water influences the impact of N on growth metrics. The three-way interaction among (year × water × N) significantly affected PH but not ST or SPH (**Table 1**).

**Table 1:** Effects of water-nitrogen interaction on plant height, stalk tick, and spike position height of maize in 2020 and 2021.

Year (Y)	Water (W)	Nitrogen (N)	Plant height (cm)	Stalk tick (mm)	Spike position height (cm)
2020	S1	N0	115.20i	11.03h	91.54e
		N100	132.33g	16.73fg	99.56d
		N200	142.17f	17.19ef	102.53cd
		N300	152.20e	17.77e	104.59c
	S2	N0	120.58h	14.83g	95.82de
		N100	163.10d	19.33d	106.63bc
		N200	189.67b	22.03bc	111.12ab
		N300	176.67c	21.00c	110.68b
	S3	N0	127.97gh	16.57fg	96.16de
		N100	173.60cd	20.20cd	107.79bc
		N200	214.33a	27.17a	119.88a
		N300	191.33b	23.77b	113.33ab
2021	S1	N0	101.067g	10.57g	84.08d
		N100	124.00e	13.60e	88.84cd
		N200	133.67de	14.27de	89.71c
		N300	144.00d	15.13d	90.73c
	S2	N0	105.47fg	12.03f	85.05d
		N100	157.33c	15.20d	91.48bc
		N200	174.00ab	18.63bc	96.98ab
		N300	169.33bc	17.33c	94.05b
	S3	N0	115.13ef	13.13ef	86.54d
		N100	161.67c	15.87d	92.50bc
		N200	182.67a	21.20a	102.57a
		N300	174.33ab	19.53b	97.69ab
ANOVA					
Y			*	*	*
W			**	**	*
N			**	**	**
Y×W			NS	NS	NS
Y×N			NS	NS	NS
W×N			**	**	*
Y×W×N			NS	NS	NS

S1: kept the soil moisture at about 40% of the field holding capacity (FHC); S2 and S3 indicated that the soil moisture was kept at about 60% and 80% of the FHC, respectively. N0: having no N; N100, N200, and N300 indicate having 100 kg N ha^-1^, 200 kg N ha^-1^ and 300 kg N ha^-1^, respectively. Mean values followed by different letters are significantly different at *p* < 0.05. *p < 0.05. **p < 0.001. NS indicates not significant.

### 4.2 Effects of water-N interaction on root growth

**Figure 2:**
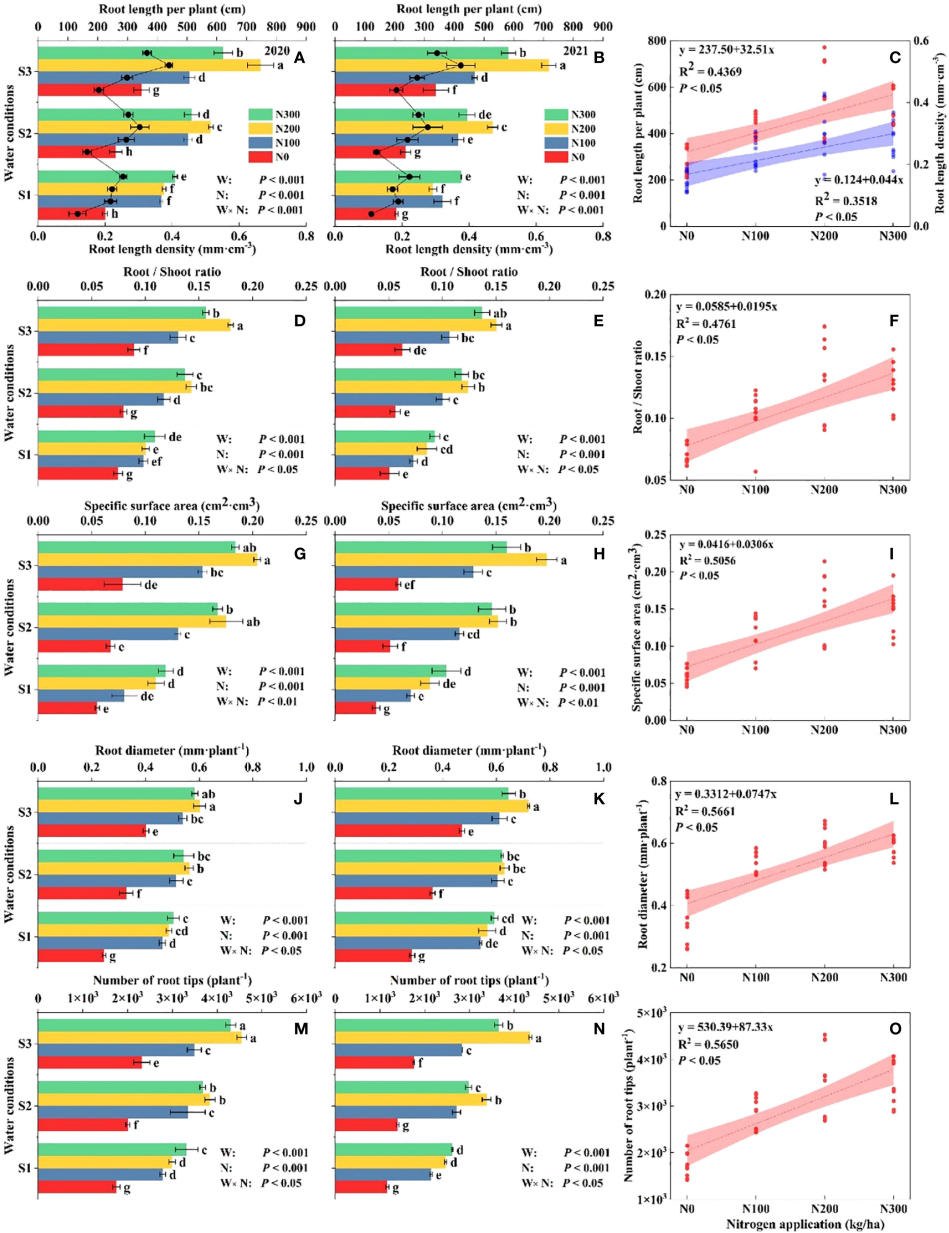
Different water conditions and N application affect the maize root. Root length (RL, **A–C**), root length density (RLD, **A–C**), root/shoot ratio (R/S, **D–F**), root-specific surface area (RSSA, **G–I**), root diameter (RD, **J–L**), and the number of root tips (RT, **M–O**) at different water-N interaction at maturity stage and relationship during 2020 and 2021.

Key growth parameters, including root length (RL), stem diameter (SD), root specific surface area (RSSA), root/shoot ratio (R/S), water content, and total dry weight, were markedly influenced by N application and water availability, highlighting the complex interactions between nutritional and environmental stress factors (**Supplementary Figure S1**; **Table S1**). Across both 2020 and 2021, RL increased with higher N levels across all water conditions, with the greatest growth observed in the N200 treatment, especially under S3 (**Figure 2**). Suggesting that adequate N availability strongly promotes root development in well-watered conditions.

The R/S ratio decreased as N increased, demonstrating a higher allocation of biomass to shoot growth at higher N levels, particularly under optimal water conditions. Similarly, N application had a positive influence on the specific area, with the highest values recorded for N200, suggesting improved root expansion and higher potential for water and nutrient absorption, which support overall plant growth. Root diameter (RD) consistently increased with higher N levels, particularly under N200, across both years, reflecting enhanced structural support and nutrient uptake capabilities. Leaf N content also increased with higher N applications, reaching peak levels under N200, a trend consistent across all water conditions, highlighting efficient N assimilation essential for photosynthesis and biomass production. Specifically, under S3-N200, RL increased by 48.75% and 24.31%, and RLD by 48.93% and 24.64% compared with S1-N200 and S2-N200, respectively (**Figures 2A, B**). Water-N interaction increased RSSA in the S3-N200 level by 44.54%, 21.95%, and 14.43% (**Figures 2G, H**), and RT by 33.46%, 25.26%, and 10.76% (**Figures 2M, N**) compared to S1-N300, S2-N300, and S3-N300 treatments, respectively.

Note: S1: kept the soil moisture at about 40% of the field holding capacity (FHC); S2 and S3 indicated that the soil moisture was kept at about 60% and 80% of the FHC, respectively. N0: having no N; N100, N200, and N300 indicate having 100 kg N ha^-1^, 200 kg N ha^-1^ and 300 kg N ha^-1^, respectively. Error bars represent the standard error, and different lowercase letters indicate significant differences at *p* < 0.05 (n = 3, Student’s *t*-test).

### 4.3 Effects of water-N interaction on root ions

**Figure 3:**
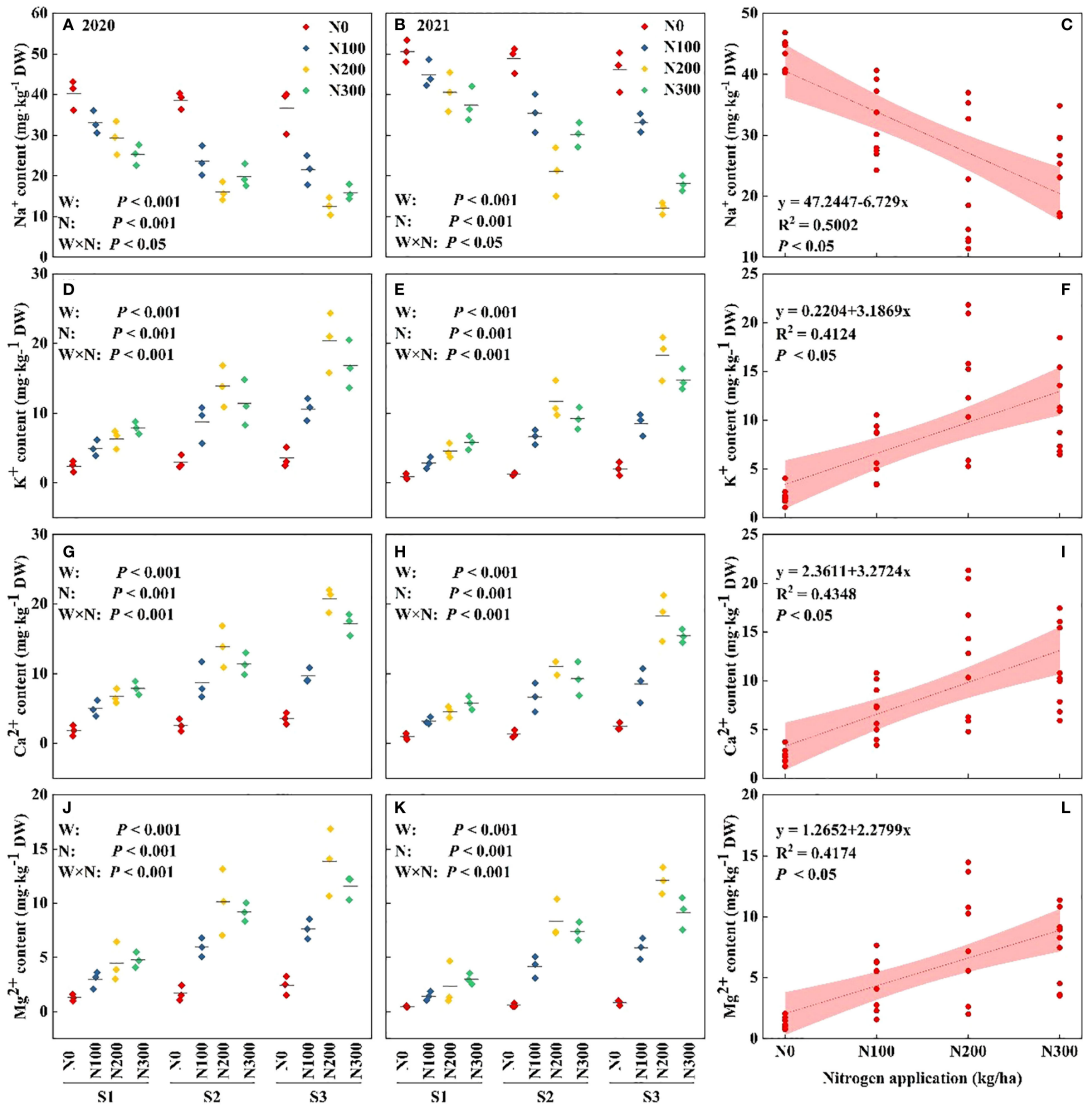
Interactive effects of water stress and N on nutrient contents in the maize root. Roots iron from **(A, B)** Na^+^, **(D, E)** K^+^, **(G, H)** Ca^2+^, and **(J, K)** Mg^2+^ in water-N interaction during 2020 and 2021. Relationship between different water conditions and Na^+^ **(C)**, K^+^ **(F)**, Ca^2+^ **(I)**, and Mg^2+^ **(L)** under different N levels during 2020-2021.

The data revealed that ion concentrations were significantly elevated in 2020 compared to 2021, suggesting annual variations in ion uptake that are potentially influenced by external environmental factors beyond the experimental conditions. The increase in water conditions from S1 to S3 resulted in a significant decrease in Na^+^ levels and an increase in K^+^, Ca^2+^, and Mg^2+^ levels, emphasizing the crucial role of water availability in controlling nutrient absorption. The Na^+^ concentrations decreased by 34.12% and 15.02% in S3, respectively, compared to S1 (**Supplementary Table S2**). Compared to S3-N300, the K^+^, Ca^2+^, and Mg^2+^ content in S3-N200 were higher by 18.22%, 16.35%, and 20.02%, respectively, and by 50.49%, 53.48%, and 48.05% in S3-N100 (**Figures 3D–L**, p < 0.001). Nitrogen fertilization in S2 water condition increased K^+^, Ca^2+^, and Mg^2+^ by 40.08%, 38.47%, and 45.21% in N200 and by 25.66%, 25.41%, and 39.04% in N300 compared to N100 (**Figures 3D–L**, p < 0.001).

N200 significantly increased the concentrations of ions K^+^, Ca^2+^, and Mg^2+^ under different water conditions. In contrast, low and lack of N levels (N0 and N100) resulted in the lowest ion levels, highlighting the crucial role of N in enhancing nutrient absorption efficiency during stressful situations. The correlation between water and N was significant (*p* < 0.05), indicating the interconnected impact of these components on nutrient dynamics (**Figures 3C, F, I, L**). Nevertheless, the intricate interaction among year, water, and N indicates that external environmental conditions also have a substantial impact on nutrient absorption, extending beyond the controlled variables of water and N. These findings highlighting the complex relationships between environmental factors and agronomic techniques in determining plant mineral nutrition, particularly under variable water availability (**Figures 3C, F, I, L**, *p* < 0.05).

Note: S1: kept the soil moisture at about 40% of the field holding capacity (FHC); S2 and S3 indicated that the soil moisture was kept at about 60% and 80% of the FHC, respectively. N0: having no N; N100, N200, and N300 indicate having 100 kg N ha^-1^, 200 kg N ha^-1^ and 300 kg N ha^-1^, respectively. Error bars represent the standard error, and different lowercase letters indicate significant differences at *p* < 0.05 (n = 3, Student’s *t*-test).

### 4.4 Effects of water-N interaction on root hormones

**Figure 4:**
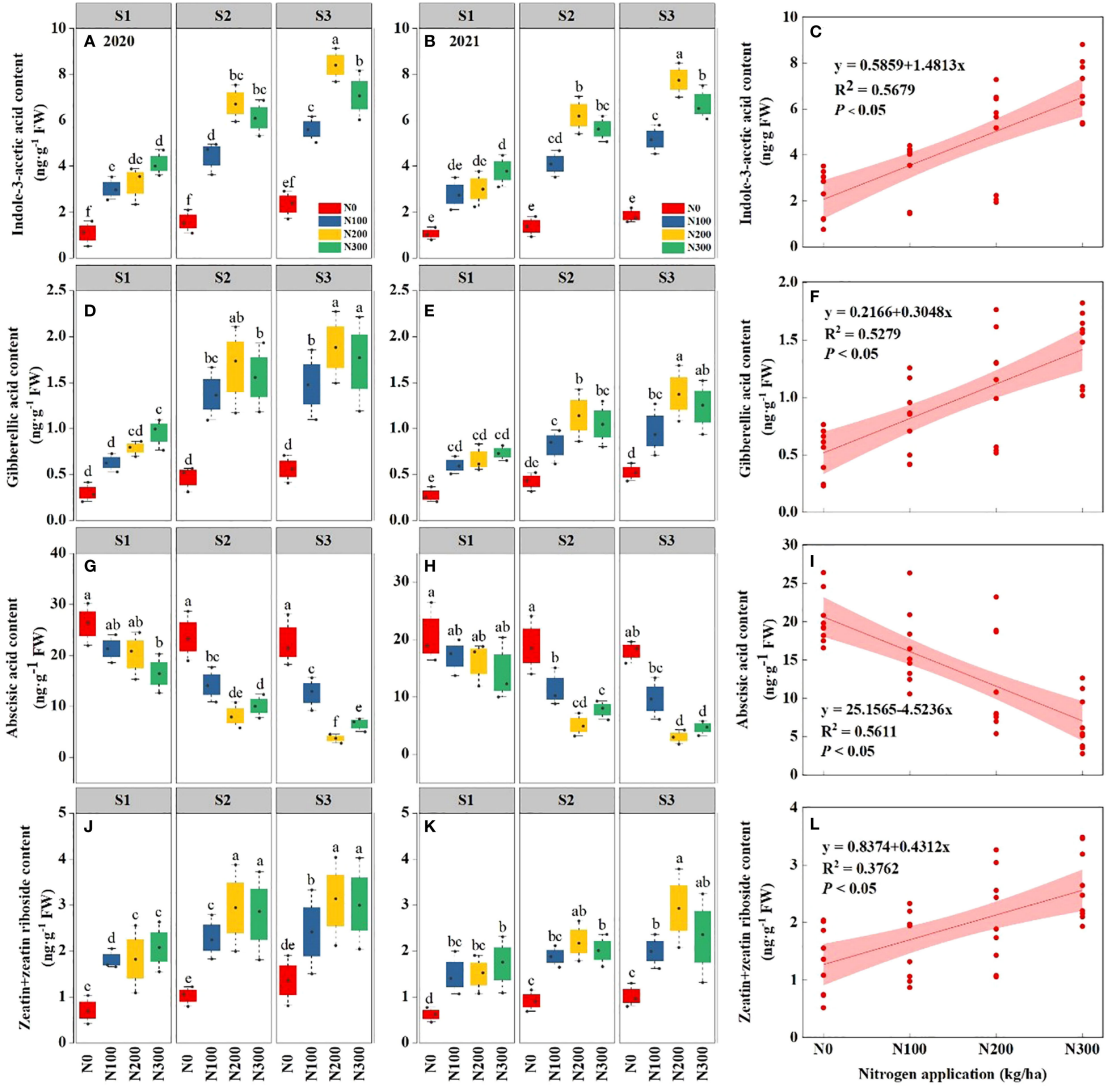
Interactive effects of water stress and nitrogen interaction on different hormones in maize roots (*Maize* L. cv. Nendan19) during 2020 and 2021. **(A, B, D, E, G, H)**, and **(J, K)** indicate the Indole-3-acetic acid (IAA), gibberellic acid (GA), abscisic acid (ABA), and zeatin+zeatin riboside (Z + ZR), respectively. Relationship between different water conditions and IAA **(C)**, GA **(F)**, ABA **(I)**, and Z + ZR **(L)** under different nitrogen levels during 2020-2021. S1: kept the soil moisture at about 40% of the field holding capacity (FHC); S2 and S3 indicated that the soil moisture was kept at about 60% and 80% of the FHC, respectively. N0: having no N; N100, N200, and N300 indicate having 100 kg N/ha, 200 kg N/ha and 300 kg N/ha, respectively. Error bars represent the standard error, and different lowercase letters indicate significant differences at *p* < 0.05 (n = 3, Student’s *t*-test).

The findings elucidate the intricate relationship between water conditions, N levels, and the hormonal equilibrium in maize roots (**Figure 4**). Across both years, increasing water levels from S1 to S3 consistently reduced ABA concentrations while increasing IAA, GA, and Z+ZR. The changes were more significant in 2021, suggesting a potential cumulative stress effect over successive years. Notably, under S3, ABA content decreased by 46.04% (9.69 ng g^-1^) and 19.01% (2.67 ng g^-1^) in 2020 and 48.17% (8.19 ng g^-1^), 18.23% (1.96 ng g^-1^) in 2021, compared with S1 and S2, respectively (**Supplementary Table S3**).

Nitrogen application, particularly N200 (2.02 g pot^-1^) markedly increased all hormones concentrations compared to low (N0 and N100) and high N dosage (N300). This suggests that sufficient N availability can alleviate certain hormonal disturbances induced by water stress. A significant interaction between water and N, particularly for IAA and ABA, highlighting the complex hormonal response to environmental pressures (**Figures 4A–C** and **Figures 4G–I**). These findings underscore the importance of managing water and N synergistically to maintain hormonal equilibrium and enhance the ability of plants to withstand stressful conditions.

### 4.5 Effects of water-N interaction on root osmoregulation substance, ROS

**Figure 5:**
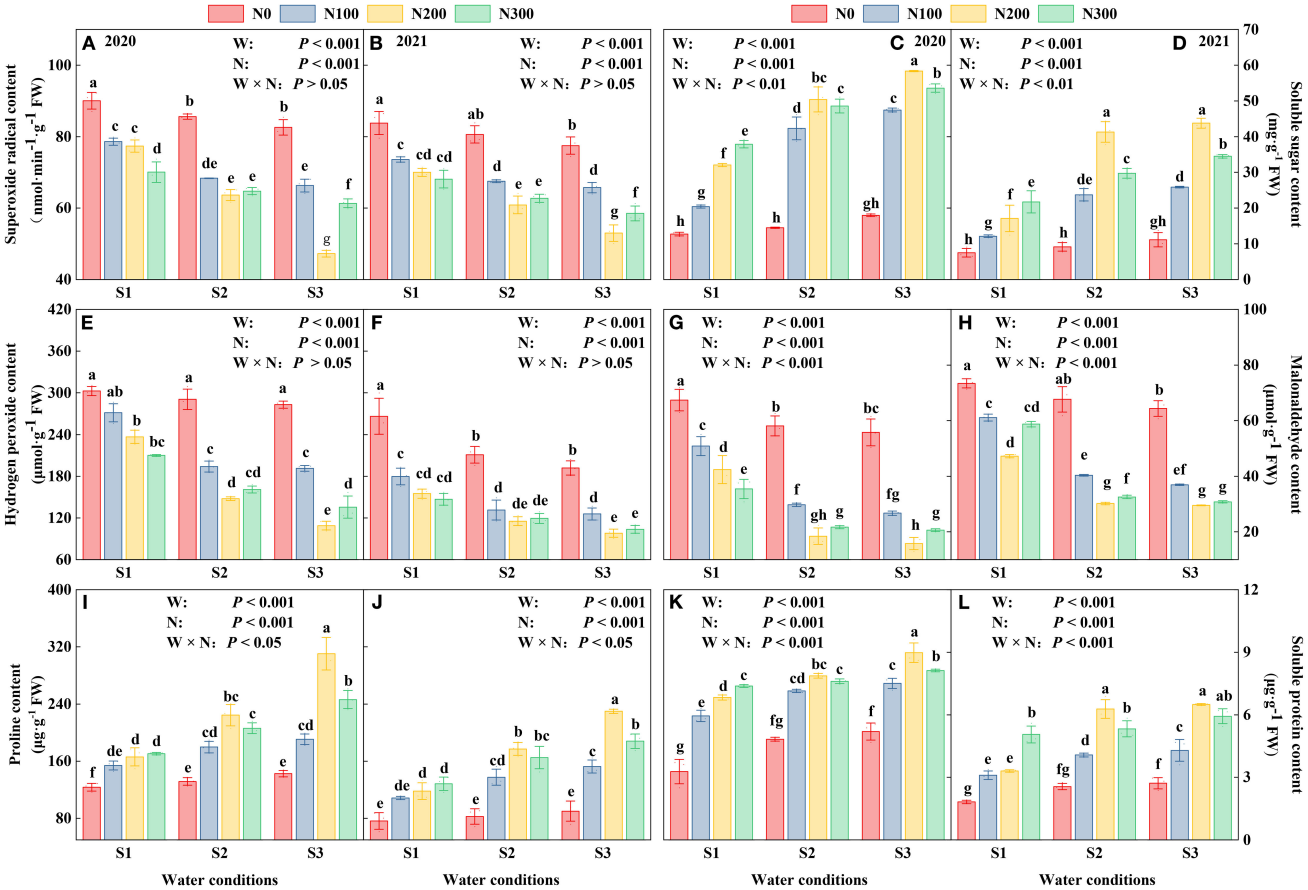
Interactive effects of water stress and nitrogen on the roots of maize (*Maize* L. cv. Nendan19) superoxide radical **(A, B)**, soluble sugar **(C, D)**, hydrogen peroxide **(E, F)**, malonaldehyde **(G, H)**, proline **(I, J)**, soluble protein **(K, L)** during 2020 and 2021.

The study highlights variations in reactive oxygen species (ROS), including O^2-^ and H_2_O_2_, along with Pro, SS, MDA, and SP, in response to different environmental conditions (**Figure 5**). From 2020 to 2021, all markers declined, suggesting either adaptive plant responses or changes in experimental settings that affect plant stress reactivity. Notably, the S3 consistently showed elevated markers, suggesting a fundamental resistance to oxidative stress. Conversely, N200 noticeably increased stress indicators, indicating a heightened metabolic activity and probable relief from stress (**Figure 5**; **Supplementary Table S4**).

Water conditions from S1 to S3 gradually decreased stress indicators, with the lowest values observed in the S1 treatment. This suggests a possible slowdown or halt in metabolic activities under severe water deficit. The synergistic effects of N levels and water conditions were especially evident in the levels of O_2_^-^, H_2_O_2_, and MDA (**Figures 5A, B, E–H**). These results demonstrate the complex relationship between nutrient availability and water conditions in controlling plant physiological responses. Additionally, these results highlight the importance of accurate N management and a comprehensive understanding of water conditions in enhancing plant resilience against environmental pressures. On average, N200 treatment reduced Proline, SS, and SP contents by 24.39-44.63%, 15.94-41.59%, and 10.11-19.62% compared to N300 and by 36.46-51.41%, 28.22-68.10%, and 23.73-41.52% compared to N100 (**Supplementary Table S4**).

Note: S1 (with soil moisture maintained at about 40% of field holding capacity); S2 and S3 indicated that kept the soil moisture at about 60% and 80% of the soil’s FHC. Error bars represent the standard error, and different lowercase letters indicate significant differences at *p* < 0.05 (n = 3, Student’s *t*-test).

### 4.6 Effects of water-N interaction on root antioxidant

**Figure 6:**
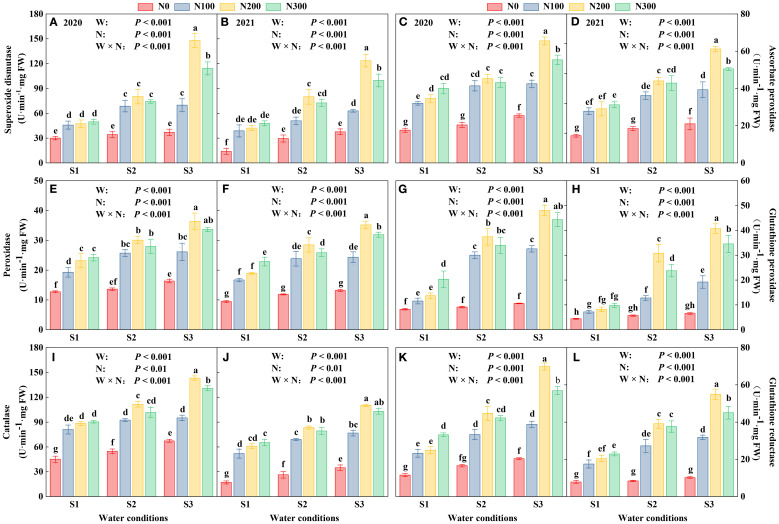
Interactive effects of water stress and N on the roots of maize (*Maize* L. cv. Nendan19) superoxide dismutase **(A, B)**, ascorbate peroxidase **(C, D)**, peroxidase **(E, F)**, glutathione peroxidase **(G, H)**, catalase **(I, J)**, glutathione reductase **(K, L)** during 2020 and 2021. S1 (with soil moisture maintained at about 40% of field holding capacity); S2 and S3 indicated that they kept the soil moisture at about 60% and 80% of the soil’s FHC. Error bars represent the standard error, and different lowercase letters indicate significant differences at *p* < 0.05 (n = 3, Student’s *t*-test).

Oxidant enzyme activities, including SOD, POD, CAT, APX, GPX, and GR, were quantified in plant tissues exposed to water conditions and varying N levels (**Figure 6**). Enzyme activities in 2020 were greater than those in 2021, suggesting a difference in oxidative stress responses or environmental factors influencing enzyme expression (**Supplementary Table S5**). The highest activities were observed under S3, with a notable increase as water condition intensified from S1 to S3, emphasizing the critical role of water condition in enhancing antioxidant defense systems. Nitrogen had a dose-dependent impact, N200 consistently leading to the highest levels of enzyme activity (**Supplementary Table S5**).

The activity of antioxidant enzymes showed significant variation due to the interplay between water and N, indicating intricate physiological responses to the environmental stressors. These findings underscore the need for tailored agronomic strategies to maximize plant health and enhance stress resistance in managing water and N levels. Under S3 conditions, the activities of antioxidant enzymes showed significant enhancements compared to S1 and S2 across all N levels. Specifically, SOD activity increased by 36.35%, 67.01%, and 54.37% at N100, N200, and N300, respectively, relative to S1, and by 10.05%, 41.01%, and 31.39% relative to S2 (**Figures 6A, B**; **Supplementary Table S5**). APX activity increased by 26.01%, 49.48%, and 32.12% over S1, and by 4.89%, 29.71%, and 19.10% over S2 at the respective N levels (**Figures 6C, D**; **Supplementary Table S5**). Similarly, CAT activity showed increases of 22.59%, 41.02%, and 33.52% over S1, and 6.05%, 23.04%, and 22.60% over S2 at N100, N200, and N300, respectively (**Figures 6I, J**; **Supplementary Table S5**).

### 4.7 Effects of water-N interaction on root non-enzyme antioxidants

**Figure 7:**
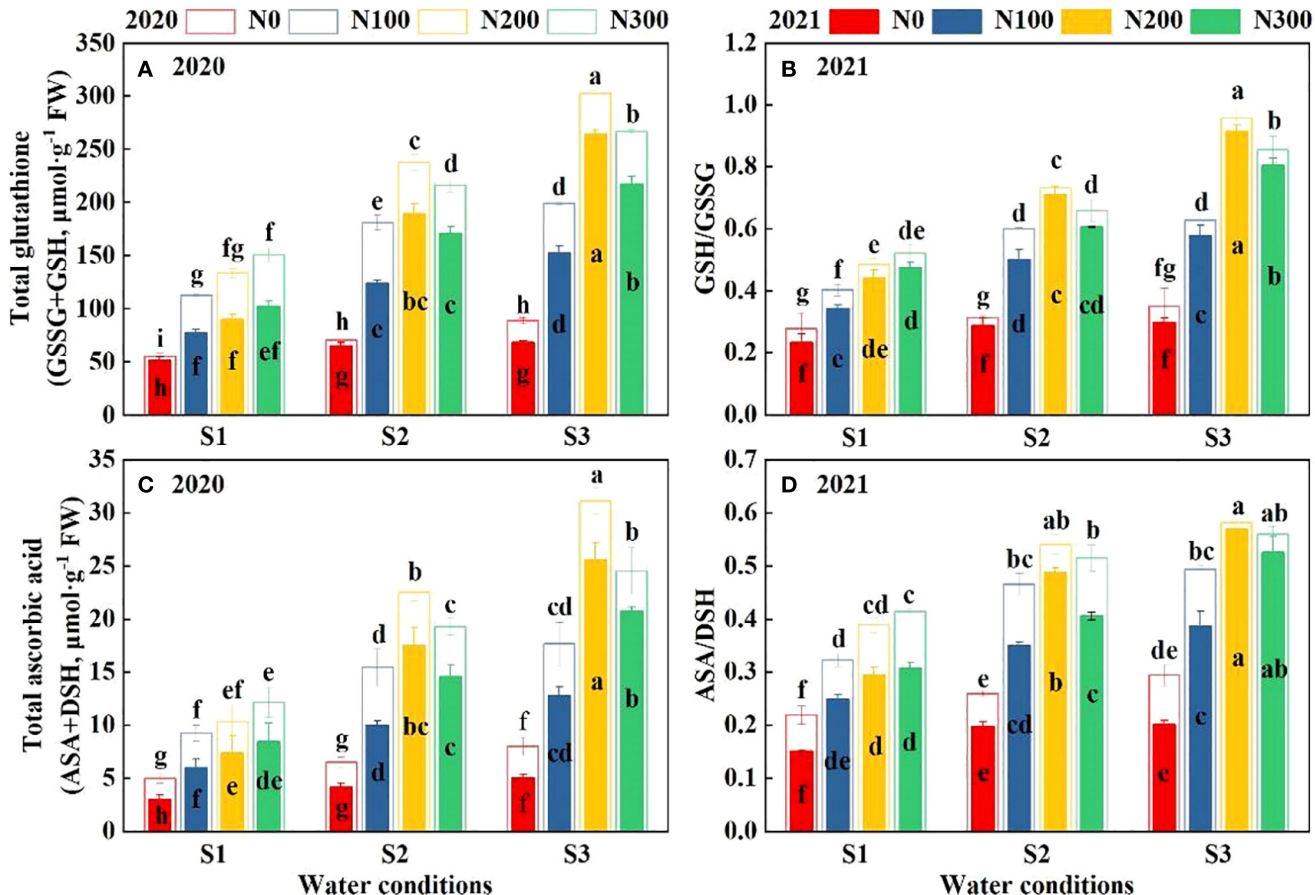
Interactive effects of water stress and N on total glutathione **(A)**, GSH/GSSG ratio **(B)**, total ascorbic acid **(C)**, and ASA/DHA ratio **(D)** content of roots in 2020 and 2021. S1: kept the soil moisture at about 40% of the field holding capacity (FHC); S2 and S3 indicated that the soil moisture was kept at about 60% and 80% of the FHC, respectively. N0: having no N; N100, N200, and N300 indicate having 100 kg N ha^-1^, 200 kg N ha^-1^ and 300 kg N ha^-1^, respectively. Error bars represent the standard error, and different lowercase letters indicate significant differences at *p* < 0.05 (n = 3, Student’s *t*-test).

The concentration of GSSG+GSH, GSH/GSSH, ASA+DSH, and ASA/DSH was significantly affected by N, water, and their interactions (**Figures 7A–D**). Over the two-year study, elevated N rates consistently enhanced GSSG+GSH and ASA+DS, with higher N rates yielding greater improvements. This highlights the crucial role of N in improving the plant’s antioxidant capacity. Notably, the N200 treatment demonstrated the highest glutathione levels among all water treatments. There was a marked increase in these levels as water conditions intensified from S1 to S3. Similarly, the ratio of GSH to GSSG was found to be highest in the N200 treatment. This indicates that plants receiving higher N levels can more efficiently control oxidative stress. The ascorbate levels exhibited a comparable trend, reaching their peak under conditions of S3-N200, suggesting that the accumulation of these crucial antioxidants is promoted by adequate N and water availability.

In 2021, although the levels of GSSG+GSH and ASA+DSH were reduced compared to 2020. Nitrogen application significantly influenced non-enzyme antioxidant levels (**Supplementary Table S6**, *p* < 0.01). Nitrogen, water, and their interactions significantly affect the GSSG+GSH, GSH/GSSH, ASA+DSH, and ASA/DSH (**Supplementary Table S6**; *p* < 0.01). The N200 treatment significantly increased antioxidant components compared with N100 and N300 across all water conditions in 2020-2021. Particularly, GSSG+GSH increased by 66.76% and 55.71% under S1, 46.60% and 31.91% under S2, and 38.31% and 14.83% under S3 (**Figure 7A**). Likewise, GSH/GSSH increased by 60.17% and 46.75% (S1), 41.23% and 29.69% (S2), 35.59% and 11.28% (S3) (**Figure 7B**). ASA+DSH increased by 73.34% and 63.83% (S1), 55.51% and 40.52% (S2), 46.48% and 19.91% (S3) (**Figure 7C**). ASA/DSH increased by 50.27% and 37.24% under S1, 29.08% and 19.98% under S2, 23.51% and 5.53% under S3, respectively (**Figure 7D**).

### 4.8 Effects of water-N interaction on biomass accumulation and partitioning and maize yield

**Figure 8:**
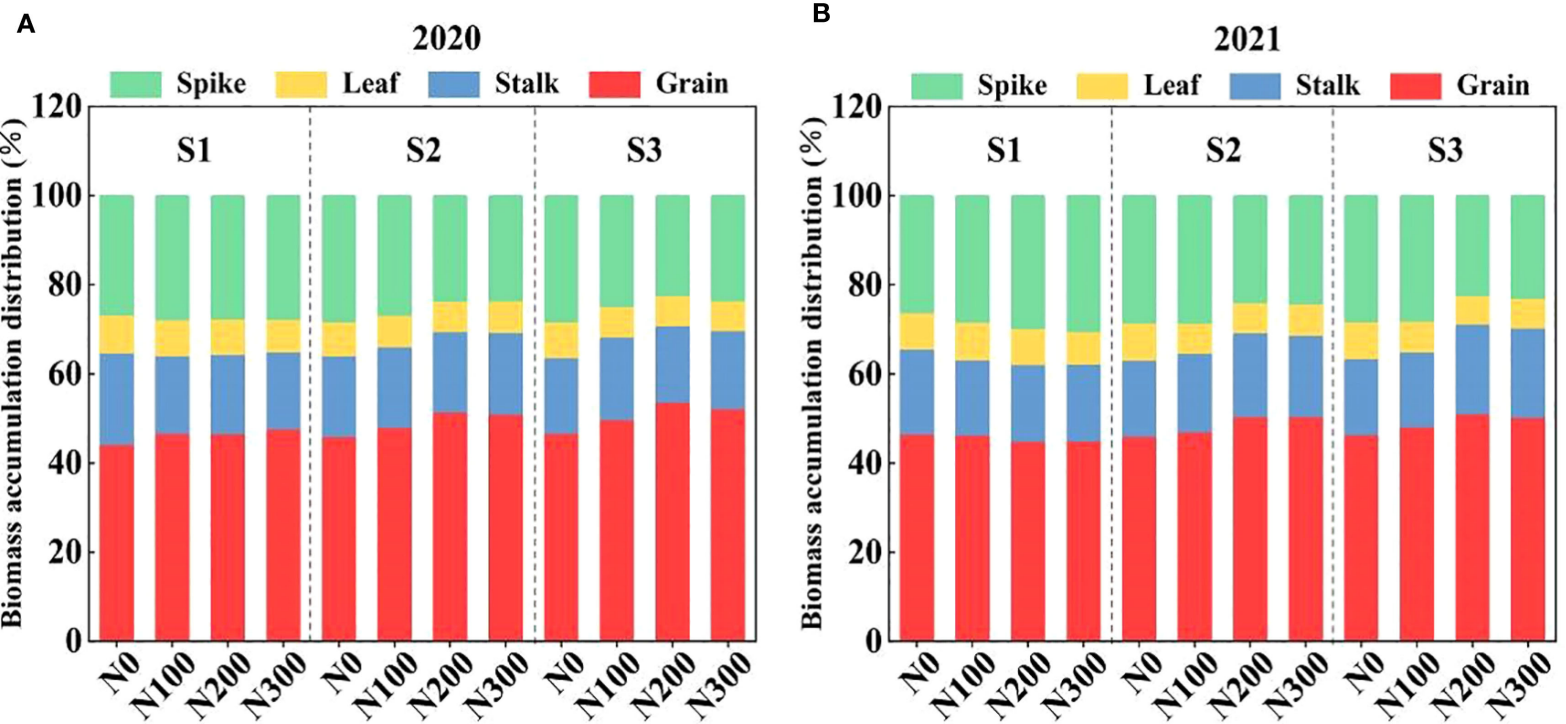
Interactive effects of water stress and N on biomass accumulation and distribution in spike, leaf, stalk, and grain of maize after maturity during 2020 **(A)** and 2021 **(B)**. S1: kept the soil moisture at about 40% of the field holding capacity (FHC); S2 and S3 indicated that the soil moisture was kept at about 60% and 80% of the FHC, respectively. N0: having no N; N100, N200, and N300 indicate having 100 kg N ha^-1^, 200 kg N ha^-1^ and 300 kg N ha^-1^, respectively.

The allocation of biomass across various plant components (spike, leaf, stalk, and grain) in 2020 and 2021 was significantly influenced by varying water conditions and N application rates (**Figure 8**). During both years, observable shift occurred in dry matter distribution from vegetative parts such as leaves and stalks, to reproductive parts, including spikes and grains, as the water condition increased from S1 to S3. This indicates that there were adaptive responses to the environmental stress.

In 2020, N200 consistently resulted in more biomass being allocated to the grain, regardless of the water treatments. These results suggest that N plays a crucial role in enhancing the reproductive performance of plants, even under varying water conditions. Conversely, N0 and N300 treatments decreased dry matter allocation in grain, especially when subjected to lower water conditions (S1). Under S2 condition, N200 treatment resulted in higher biomass accumulation by 34.51% and 57.98% (spike), 41.97% and 56.30% (leaf), 31.59% and 48.01% (stalk), 30.66% and 45.01% (grain) compared to N100 and N300, respectively. Similarly, N200 treatment increased biomass accumulation by 42.23% and 61.29% (spike), 43.65% and 61.23% (leaf), 28.29% and 51.93% (stalk), and 29.78% and 48.03% (grain), respectively, compared to N100 and N300 under S3 (**Figure 8A**). The biomass accumulation was increased by 38.95% and 65.61% (spike), 48.82% and 66.26% (leaf), 38.93% and 50.25% (stalk), and 35.77% and 54.23% (grain) in S3-N200 compared to the S3-N100 and S3-N300 treatments (**Figure 8B**).

In 2020, the N200 treatment consistently yielded the highest values for each parameter, including a greater number of rows per ear, grain number per row, 100-grain weight, total grains per ear, and yield per plant (**Table 2**). Conversely, N0 resulted in the lowest values for the above parameters, emphasizing the crucial importance of optimizing water-N in promoting strong ear growth and grain filling. As the level of water increased, and there was a corresponding rise in all the measured metrics. S3 exhibited the most significant values, but S1 had the lowest values. The observed pattern was consistent across both N treatments and years, highlighting the cumulative impact of increased water availability on crop productivity. Under S3-N200 treatments, improvements were noted in 100-grain weight (2020: 17.76%, 8.64% and 4.07%), and (2021: 14.71%, 8.99%, and 3.96%), Grains per ear (2020: 17.94%, 10.07%, and 3.37%), and (2021: 17.32%, 7.09%, and 3.54%), yield (2020: 32.64%, 18.03%, and 7.39%), and (2021: 29.45%, 15.49%, and 7.42%) compared with S1-N300, S2-N300, and S3-N300 treatments (**Table 2**). These findings suggest that the management of water and N is crucial for optimizing crop outcomes in varying environmental conditions. Occasionally, the non-significant interactions among year, water, and N (Y × W × N) suggest that the separate impacts of year, water, and N may sometimes be more prominent than their combined contributions (*p* < 0.01).

**Table 2:** Effects of water-N interaction on row per ear, grain per row, 100-grain weight, grain per ear, and yield of maize.

Year (Y)	Water (W)	Nitrogen (N)	Row3per ear	Grain per row	100-grain weight (g)	Grains per ear	Yield (g plant^-1^)
2020	S1	N0	18.33 g	14.67 g	26.29 h	268.67 i	70.64 i
		N100	19.33 ef	15.00 fg	27.82 gh	290.00 hi	80.63 hi
		N200	19.67 de	15.33 fg	28.35 fg	301.67 gh	85.54 gh
		N300	19.67 de	15.67 ef	29.69 ef	308.00 fg	91.41 fg
	S2	N0	18.67 fg	15.00 fg	26.66 gh	280.00 hi	74.65 i
		N100	20.00 cd	16.00 de	30.17 de	320.00 ef	96.54 ef
		N200	21.00 bc	16.67 bc	33.83 b	349.67 bc	118.21 bc
		N300	20.67 bc	16.33 cd	32.98 bc	337.33 cd	111.24 cd
	S3	N0	19.00 ef	15.00 fg	27.19 gh	285.00 hi	77.45 hi
		N100	20.33 cd	16.00 de	31.54 cd	325.33 de	102.63 de
		N200	21.67 a	17.33 a	36.10 a	375.33 a	135.71 a
		N300	21.33 ab	17.00 ab	34.63 ab	362.67 ab	125.67 b
2021	S1	N0	18.00 d	14.00 e	24.87 i	252.00 d	62.57 h
		N100	18.33 d	14.33 de	27.35 gh	263.00 d	71.71 gh
		N200	18.67 cd	14.67 de	28.05 fg	273.67 cd	76.74 fg
		N300	18.67 cd	15.00 cd	29.29 ef	280.00 cd	82.04 ef
	S2	N0	18.33 d	14.33 de	25.34 hi	262.3 d	66.67 gh
		N100	19.00 bc	15.67 bc	29.86 de	297.33 bc	89.16 de
		N200	19.67 ab	16.00 bc	32.22 bc	314.67 ab	101.47 bc
		N300	19.67 ab	16.00 bc	31.25 cd	314.67 ab	98.27 cd
	S3	N0	18.33 d	14.33 de	26.65 hi	263.00 d	69.91 gh
		N100	19.00 bc	15.67 bc	30.54 cd	298.00 bc	90.80 de
		N200	20.33 a	16.67 a	34.34 a	338.67 a	116.29 a
		N300	20.00 ab	16.33 ab	32.98 ab	326.67 ab	107.66 ab
ANOVA							
Y			**	NS	**	**	**
W			**	**	**	**	**
N			**	**	**	**	**
Y×W			NS	NS	**	NS	**
Y×N			NS	NS	**	**	**
W×N			*	NS	**	**	**
Y×W×N			NS	NS	NS	NS	NS

CK: kept the soil moisture at about 75-80% of the soil’s field holding capacity (FHC); S1, S2, and S3 indicated that they kept the soil moisture at about 65-70%, 55-60%, and 45-50% of the soil’s FC, respectively. Mean values followed by different letters are significantly different at *p* < 0.05. *p < 0.05. **p < 0.01. NS indicates not significant.

### 4.9 Correlations between variables

**Figure 9:**
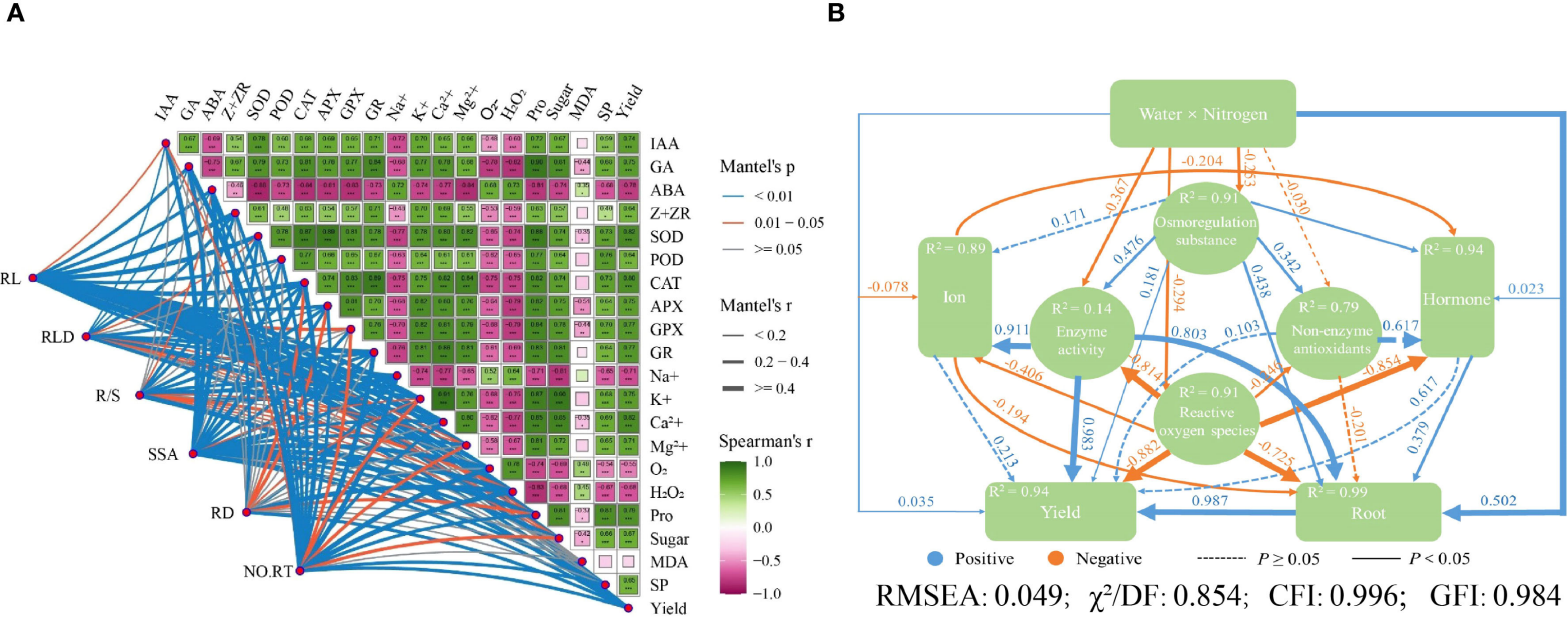
**(A)** Correlation analysis of enzyme activity, ion, hormone, root growth, and maize yield. Green represents a positive correlation, and pink represents a negative correlation. The darker the color, the stronger the correlation, and vice versa. The numbers in the boxes represent the correlation coefficients. RL, Root length; RLD, root length density; R/S, root/shoot ratio; RSSA, root specific surface area; RD, root diameter; NO.RT, number of root of tips; IAA, Indole-3-acetic acid; GA, gibberellic acid; ABA, abscisic acid; zeatin+zeatin riboside, Z+ZR; SOD, superoxide radical; POD, peroxidase; CAT, catalase; APX, ascorbate peroxidase; GPX, glutathione peroxidase; GR, glutathione reductase; O^2-^, superoxide anion; H_2_O_2_, hydrogen peroxide; Pro, proline; MDA, malonaldehyde; SP, soluble protein. **(B)** Structural equation modeling results based on the correlations among different water-N interactions (*P* = 0.426). The path coefficient represents the direction and intensity of the linear relationship between latent variables, and R^2^ represents the percentage of variation explained by other variables. Numbers near the boxes indicate the amount of variance explained by the model (R^2^).

The positive correlations were observed between root traits, such as RL, root length density, R/S ratio, RSSA, root diameter (RD), number of root tips, and some biochemical and physiological parameters. These include IAA (R^2^ = 0.54-0.78, *p* < 0.01), GA (R^2^ = 0.67-0.90, *p* < 0.01), Z+ZR (R^2^ = 0.40-0.70, *p* < 0.05), and enzymes such as SOD (R^2^ = 0.48-0.89, *p* < 0.05), POD (R^2^ = 0.61-0.77, *p* < 0.01), CAT (R^2^ = 0.73-0.89, *p* < 0.01), APX (R^2^ = 0.64-0.82, *p* < 0.01), GPX (R^2^ = 0.70-0.84, *p* < 0.01), and GR (R^2^ = 0.64-0.86, *p* < 0.01). Positive correlations were found with K^+^ (R^2^ = 0.68-0.91, *p* < 0.01), Ca^2+^ (R^2^ = 0.69-0.85, *p* < 0.01), Mg^2+^ (R^2^ = 0.65-0.85, *p* < 0.01), Pro (R^2^ = 0.72-0.90, *p* < 0.01), sugar (R^2^ = 0.57-0.90, *p* < 0.01), SP (R^2^ = 0.40-0.81, *p* < 0.05) and yield (R^2^ = 0.65-0.82, *p* < 0.01; **Figure 9A**). Conversely, ABA, Na^+^, O_2_^-^, and H_2_O_2_ had a strong negative correlation with these parameters, whereas MDA content showed a lower negative correlation with root growth but was strongly negatively correlated with other parameters (**Figure 9A**).

Structural equation modeling (SEM) revealed the possible pathways by which root growth and morphology influence yield under water-N interaction, aiming to reduce the interactions among causal factors during 2020-2021 (**Figure 9B**). The model showed the best fit to the data at *P* = 0.426. In 2020-2021, water-N interaction influenced maize root growth, causing changes in osmoregulation substances, enzyme activity, reactive oxygen species, ion accumulation, hormones, and maize yield (**Figure 9B**). The model provided an excellent fit, explaining 94% variation in maize yield, 89% in ion accumulation, 94% in hormone regulation, and 91% in ROS modulation through root traits.

## 5 Discussion

Maize root distribution depends on the form of phenotypes, N concentration supplied, and water conditions. Water-N interaction increased the root parameters (RL, RLD, R/S, RSSA, RD, and RT) in 2020 and 2021 (**Figure 2**), as there were significant increases in the growth and development of roots (Zhang G, et al., 2021). The interaction between water and N increased RLD at the R6 growth stage, resulting in compensatory root growth in response to water stress (Qi et al., 2012). The root system searches for N to meet the required demand by increasing its length, number of root tips, and surface area in N-deficient conditions during the growth stage (Liu et al., 2017). Consequently, root formation also depends on soil-related properties such as soil texture, moisture content, and soil nutrients (Adu et al., 2017; De-la-Fuente et al., 2020).

### 5.1 Water-N interaction responsiveness is related to fast root growth

The root system comprised the primary, seminal, and shoot-borne nodal roots that form in whorls initiated below and above ground, as well as lateral roots in all axial root classes. Furthermore, nodal roots emerge acropetally in whorls through development either belowground or aboveground (Dowd et al., 2020). These nodal roots comprise the bulk of the axial root system, with their lateral roots playing a pivotal role in water and nutrient uptake (Schneider et al., 2020). These nodal root positions influence size-related traits, such as diameter and number (York et al., 2013). Previous research reported that RLD is essential for modeling water and nutrient movement in the vadose zone and root-shoot-atmosphere interactions (Shi et al., 2020). Similarly, RLD is the most suitable parameter to describe water uptake by plant roots compared with other root traits (Ng et al., 2019). The water and nutrient uptake increases with greater root length than root mass, and a higher specific root length tended to lead to greater plasticity in root growth and a greater physiological capacity, but shorter root longevity (Schneider et al., 2020).

The lower soil water conditions in the pots induced the roots to explore to find nutrient material, facilitating lateral and aerial root development, which explains why RL and RLD were significantly higher for roots between N concentrations with S1 than S2 (Fenn et al., 2013). We found that RT, RD, and RSSA under different water conditions were higher under N200 than N300, N100, and N0 due to soil water depletion varying rapidly in the higher N compared to the lower, and no N treatments, where the root system failed to fulfill the N requirements of the crop. Thus, suitable water conditions promote the strong growth of roots near the N supply, increase the R/S ratio (**Figure 2**), and even improve MGP, such as PH, ST, and EPH (**Table 1**). However, a lack of N increases the root length in the non-fertilizer strip to search for nutrient absorption and utilization (Su et al., 2015). Our results showed that S3-N200 significantly promoted the root length, root/shoot ratio, root-specific surface area, root diameter, and number of root tips. The maize growth performance in the roots developed under S3-N200 and S3-N300 showed no noticeable difference. In addition, the greater amounts of water and nutrients absorbed by the lateral and aerial roots were stored in the plants to enhance the growth of the above-ground parts (Ma et al., 2013).

### 5.2 Osmotic regulation and hormones in root growth ensure suitable water-N interaction responsiveness

The effect of the root system in improving nutrient absorption under water deficit conditions, ultimately leads to improving water-root-plant relations (Basirat et al., 2019). The application of N levels decreased IAA, GAs, and Z+ZR in 2020-2021, particularly under S1 water conditions (**Figure 4**). This implies that the root system was established to absorb a large amount of soil moisture and nutrients, which is consistent with the results that showed that water stress reduced root growth (**Figure 2**). The structural equation model revealed that the endogenous hormones enhanced the growth and distribution of roots (**Figure 9**), regulated root senescence and nutrient and water transport, which in turn delayed natural death and increased root vigor (Jia et al., 2018). Appropriate soil moisture enables roots to search for water in the soil to meet plant developmental needs (Elhani et al., 2019). In addition to keeping soil moist, mucilage can help to alter the redistribution of water within the soil pore space during the drying cycle. Thus, maintaining high fluid connectivity within the soil pore space reduces ABA in the root system and promotes growth (Benard et al., 2021). The ABA in S1, S2, and S3 water conditions was slightly lower under N200 than under other N levels, thereby indicating that the root system could increase ABA production despite reduced water conditions. Our result showed that the ABA content in S3 was lower than in S1 and S2 during 2020-2021. The IAA, GAs, and Z+ZR decreased progressively with decreasing water content, as described by Nelissen et al. (2018). On the other hand, the ABA content in N0 treatment remained highest in 2020 or 2021, likely due to a lack of nutrient supply (**Figures 4G–I**). In contrast, root development under sufficient water and N treatments resulted in a gradual decrease in ABA accumulation in roots, where the S3-N200 and S3-N300 interactions played a significant role in contributing to the decrease in ABA content.

The root sheath is a key factor in plant nutrient uptake and water absorption, as it plays an essential role in enhancing the efficiency of nutrient acquisition from the soil, reflected by the ions that promote root growth (Dong et al., 2023). Under different water conditions, N levels have various effects (promotion or inhibition), not only on K^+^, Ca^2+^, and Mg^2+^, but also on Na^+^. (**Figure 3**). From the 2020–2021 mean value, we found no significant difference in N levels between N200 and N300, probably because the water was fully absorbed and utilized. As a result of changing moisture conditions, which enhanced the lateral root and aerial root due to the compensating effect of root growth, S3 was significantly better than S1 and S2. The K^+^, Ca^2+^, and Mg^2+^ in roots were positively correlated with N level, water conditions, and water-N interaction, but there was no significant water-N interaction in Na^+^. This suggests that it impacts water availability and hydraulic contact (Benard et al., 2019). The root ions possess unique biological properties that may influence the growth of the root system (Wen et al., 2021). These results are consistent with a previous demonstration that osmotically active chemicals are necessary for maintaining the development and growth of crop root cells (Kim et al., 2010).

### 5.3 Suitable water-N interaction anti-aging senescence of the root system

The maize root accumulates large amount of ROS, inducing a variety of scavengers and non-enzymatic low-molecular-weight metabolites to counteract oxidative damage under different water conditions, and even under water deficit (Mittler et al., 2022). A higher N application contributes to the accumulation of ROS in roots, particularly under a gradual increase in moisture content. Meanwhile, the root contains various organic compounds, including sugars, organic acids, organic chelators, and enzymes, which are secreted by both roots and microorganisms (Chen et al., 2022; Opoku et al., 2022). These compounds play a crucial role in enhancing the mobility of nutrients in the soil and the exchange capacity (Sharma et al., 2021). We found that the O_2_^-^, H_2_O_2_, and MDA values under S3 in the N200 level were significantly lower than those under the other water conditions during 2020 and 2021, mainly because the root system absorbed large amounts of soil nutrients and moisture. In osmoregulation, substances such as proline, soluble sugar, and soluble protein showed an opposite trend to ROS, whether under the same water conditions or N levels. Therefore, the osmoregulation substance was increased at the S3-N200 treatments. In contrast, when the maize root was treated with S1 and S2 water conditions, the levels of proline, soluble sugar, and soluble protein in N200 and N300 consistently remained at a lower level, which may have been associated with less water, as it accumulates ROS in the root systems. The N may not be fully dissolved under S1 and S2 water conditions; therefore, the root system did not meet the corresponding water and nutrient requirements due to water stress treatment. Compared to other water conditions, the water and nutrients in the roots of S3 were rapidly depleted, forcing the roots to develop incompletely and affecting maize growth (Wang et al., 2018).

Maximum root enzymatic and nonenzymatic antioxidants occurred at S3-N200 treatment in both years. The SOD and CAT were highest in different water-N interaction, followed by APX and GR, and the lowest value was measured in GPX and POD. Previous studies showed that SOD and CAT with different metal cofactors reside in the apoplast, cytosol, chloroplasts, mitochondria, nuclei, and peroxisomes, in which they process O_2_^-^ to H_2_O_2_ and also engage in H_2_O_2_ dismutation (Dvořák et al., 2020). These enzymes have been overexpressed individually or in combinations in various plant species, resulting in an enhanced performance under water stress conditions, such as preventing excessive levels of intracellular ROS, catalyzing the decomposition of H_2_O_2_ (Gomez et al., 2019). In the present study, the total glutathione during the S3-N200 treatment in 2020 and 2021 were 302.81μmol·g^-1^ FW and 263.88 μmol·g^-1^ FW, respectively, and was significantly higher than other water conditions (*p* < 0.01; **Figures 7A–C**). Under the same water condition, the N200 treatment resulted in higher GSSG/GSH and ASA/DSH ratios than the N0, N100, and N300 treatments. By contrast, if the same N application, the GSSG/GSH, and ASA/DSH gradually decreased with the decrease of moisture, and the lowest ratio was in S1 water conditions (**Figure 7B–D**). Thus, limited water availability is one of the most restrictive factors that affect the combined action of enzymatic and non-enzymatic antioxidants that regulating the growth of maize roots (Waltz, 2014). Very often, drought spells are accompanied by N deficiency, which critically exacerbates the adverse drought effects; thus, water was not the limiting factor for maize growth (Wu et al., 2022). S3-N200 and S3-N300 treatments can significantly alleviate root senescence; however, the difference was not significant.

### 5.4 Optimizing water and N management improves maize productivity

The premise of increasing grain yield is to enhance biomass quality, which is influenced by various factors, including nutrient availability, drought, flooding, and soil erosion. The dry matter accumulation and yield were closely related to the degree of an ideal root structure, to capture the water and N in the soil (Lynch, 2019). Correlation analysis showed that root morphology was significantly correlated with yield (**Figure 9A**), indicating that water-N interaction influenced maize agronomic traits (**Table 2**) and BAD (**Figure 8**). The AMOS structural equation modeling further showed that the water-N interaction can influence yield by affecting root morphology (**Figure 9A**). Under the S3 water conditions, N200 was most suitable because it promoted the growth and distribution of RL, RLD, root/shoot ratio, root specific surface area, RD, and the number of root tips. Moreover, increasing the root ions and hormones to promote biomass accumulation in the aboveground plant parts increases maize yield (**Figure 9B**). Therefore, adjusting the root development and distribution by regulating water-N interactions effectively improves water and fertilizer use efficiency, promoting biomass accumulation and increasing grain yield.

### 5.5 Limitations and implications

This study offers valuable insight into water–nitrogen interaction in maize, but it is limited to a single genotype and specific climatic conditions. Such specificity may restrict the generalizability of the findings to other environments. Additionally, soil biological factors, particularly microbial interactions, were not considered, although they may significantly influence root development and nutrient uptake. The precise water-nitrogen regulation used in this study also involves labor-intensive practices, which could hinder large-scale implementation. Nevertheless, the results highlight the potential of optimized irrigation and nitrogen management to improve productivity, conserve resources, and minimize environmental impacts on semi-arid regions facing water scarcity.

## 6 Conclusions

Optimizing water and nitrogen input significantly improves maize root architecture, nutrient uptake, and dry matter accumulation. The S3-N200 treatment achieved comparable yield and root performance to S3-N300 while reducing nitrogen use, offering a more resource-efficient and environmentally sustainable alternative. This strategy enhances maize productivity while conserving water and nitrogen, making it particularly suitable for cold semi-arid regions where water availability is declining.

Looking ahead, the future research should build on these findings by conducting multi-genotypic trials across diverse agro-climatic zones in China. Further investigation into soil microbial communities and their interaction with maize under varying water-nitrogen regimes will offer deeper insights into long-term soil health and nutrient cycling. Such integrative approaches will help develop a robust, climate–resilient crop management systems that promote sustainable agriculture and ensures food security in water-limited environments.

## Data Availability

The original contributions presented in the study are included in the article/**Supplementary Material**. Further inquiries can be directed to the corresponding author.

## References

[B1] Adu M. O. Asare P. A. Yawson D. O. Ackah F. K. Amoah K. K. Nyarko M. A.. (2017). Quantifying variations in rhizosheath and root system phenotypes of landraces and improved varieties of juvenile maize. Rhizosphere-Neth. 3, 29–39. doi: 10.1016/j.rhisph.2016.12.004

[B2] Ahmad S. Wang G.-Y. Muhammad I. Zeeshan M. Zhou X.-B. (2022). Melatonin and KNO3 application improves growth, physiological and biochemical characteristics of maize seedlings under waterlogging stress conditions. Biology 11 (1), 99., PMID: 35053096 10.3390/biology11010099 PMC8773118

[B3] Aksu G. Altay H. (2020). The effects of potassium applications on drought stress in sugar beet. Sugar Tech. 22, 1092–1102. doi: 10.1007/s12355-020-00851-w

[B4] Araus J. L. Slafer G. A. Royo C. Serret M. D. (2008). Breeding for yield potential and stress adaptation in cereals. Crit. Rev. Plant Science. 27, 377–412. doi: 10.1080/07352680802467736

[B5] Basirat M. Mousavi S. M. Abbaszadeh S. Ebrahimi M. Zare-banadkouki M. (2019). The rhizosheath: a potential root trait helping plants to tolerate drought stress. Plant Soil. 445, 565–575. doi: 10.1007/s11104-019-04334-0

[B6] Benard P. Schepers J. R. Crosta M. Zarebanadkouki M. Carminati A. (2021). Physics of viscous bridges in soil biological hotspots. Water Resour. Res. 57, e2021WR030052. doi: 10.1029/2021WR030052

[B7] Benard P. Zarebanadkouki M. Carminati A. (2019). Physics and hydraulics of the rhizosphere network. J. Plant Nutr. Soil Sci. 182, 5–8. doi: 10.1002/jpln.201800042

[B8] Chen Y. Yao Z. Sun Y. Wang E. Tian C. Sun Y.. (2022). Current studies of the effects of drought stress on root exudates and rhizosphere microbiomes of crop plant species. Int. J. Mol. Sci. 23, 2374. doi: 10.3390/ijms23042374, PMID: 35216487 PMC8874553

[B9] De-la-Fuente C. C. Simonin M. King E. Moulin L. Bennett M. J. Castrillo G.. (2020). An extended root phenotype: the rhizosphere, its formation and impacts on plant fitness. Plant J. 103, 951–964. doi: 10.1111/tpj.14781, PMID: 32324287

[B10] Dong J. Norman J. V. Žárský V. Zhang Y. (2023). Plant cell polarity: The many facets of sidedness. Plant Physiol. 193, 1–5. doi: 10.1093/plphys/kiad436, PMID: 37565502 PMC10469367

[B11] Dowd W. T. Braun D. M. Sharp R. E. (2020). Maize lateral root developmental plasticity induced by mild water stress. II: Genotype-specific spatio-temporal effects on determinate development. Plant Cell Environ. 43, 2409–2427. doi: 10.1111/pce.13840, PMID: 32644247

[B12] Dvořák P. Krasylenko Y. Ovečka M. Basheer J. Zaplová V. Šamaj J.. (2020). FSD1: a plastidial, nuclear and cytoplasmic enzyme relocalizing to the plasma membrane under salinity. bioRxiv 2020.2003. 2024.005363.

[B13] Elhani S. Haddadi M. Csákvári E. Zantar S. Hamim A. Villányi V.. (2019). Effects of partial root-zone drying and deficit irrigation on yield, irrigation water-use efficiency and some potato (*Solanum tuberosum* L.) quality traits under glasshouse conditions. Agric. Water Manage. 224, 105745. doi: 10.1007/s00271-009-0159-y

[B14] Erenstein O. Jaleta M. Sonder K. Mottaleb K. Prasanna B. M. (2022). Global maize production, consumption and trade: trends and R&D implications. Food Secur. 14 (5), 1295–1319.

[B15] FAO (2020). FAOSTAT Database (Rome: FAO).

[B16] Fenn M. E. Ross C. S. Schilling S. L. Baccus W. D. Larrabee M. A. Lofgren R. A. (2013). Atmospheric deposition of nitrogen and sulfur and preferential canopy consumption of nitrate in forests of the Pacific Northwest, USA. For. Ecol. Manage. 302, 240–253. doi: 10.1016/j.foreco.2013.03.042

[B17] Gomez R. Vicino P. Carrillo N. Lodeyro A. F. (2019). Manipulation of oxidative stress responses as a strategy to generate stress-tolerant crops. From damage to signaling to tolerance. Crit. Rev. Biotechnol. 39, 693–708. doi: 10.1080/07388551.2019.1597829, PMID: 30991845

[B18] Hu B. Chu C. (2020). Nitrogen-phosphorus interplay: old story with molecular tale. New Phytol. 225, 1455–1460. doi: 10.1111/nph.16102, PMID: 31400226

[B19] Jia Q. Xu Y. Ali S. Sun L. Ding R. Ren X.. (2018). Strategies of supplemental irrigation and modified planting densities to improve the root growth and lodging resistance of maize (*Zea mays* L.) under the ridge-furrow rainfall harvesting system. Field Crop Res. 224, 48–59. doi: 10.1016/j.fcr.2018.04.011

[B20] Kim T. H. Böhmer M. Hu H. Nishimura N. Schroeder J. I. (2010). Guard cell signal transduction network: Advances in understanding abscisic acid, CO_2_, and Ca^2+^ signaling. Annu. Rev. Plant Biol. 61, 561–591. doi: 10.1146/annurev-arplant-042809-112226, PMID: 20192751 PMC3056615

[B21] Liu Z. Zhu K. Dong S. Liu P. Zhao B. Zhang J. (2017). Effects of integrated agronomic practices management on root growth and development of summer maize. Euro. J. Agron. 84, 140–151. doi: 10.1016/j.eja.2016.12.006

[B22] Lynch J. P. (2015). Root phenes that reduce the metabolic costs of soil exploration: Opportunities for 21st century agriculture. Plant Cell Environ. 38, 1775–1784. doi: 10.1111/pce.12451, PMID: 25255708

[B23] Lynch J. P. (2019). Root phenotypes for improved nutrient capture: an underexploited opportunity for global agriculture. New Phytol. 223 (2), 548–564. 30746704 10.1111/nph.15738

[B24] Ma Q. Zhang F. Rengel Z. Shen J. (2013). Localized application of NH4+-N plus P at the seedling and later growth stages enhances nutrient uptake and maize yield by inducing lateral root proliferation. Plant Soil. 372 (1), 65–80

[B25] Maggio A. Zhu J. K. Hasegawa P. M. Bressan R. A. (2006). Osmogenetics: aristotle to arabidopsis. Plant Cell. 18, 1542–1557. doi: 10.1105/tpc.105.040501, PMID: 16809814 PMC1488915

[B26] Mittler R. Zandalinas S. I. Fichman Y. Breusegem F. V. (2022). Reactive oxygen species signalling in plant stress responses. Nat. Rev. Mol. Cell Bio. 23, 663–679. doi: 10.1038/s41580-022-00499-2, PMID: 35760900

[B27] Muhammad I. Fahad S. Khalofah A. Zheng B. Shen W. (2025). Melatonin enhances antioxidant defense systems and stress tolerance in plants under variable environmental conditions. Rice. 18, 70. doi: 10.1186/s12284-025-00825-0, PMID: 40690078 PMC12279687

[B28] Muhammad I. Yang L. Ahmad S. Farooq S. Khan A. Zeeshan M.. (2022a). Low irrigation water improves biomass saccharification, photosynthetic pigments of maize, and minimizes nitrate nitrogen leaching. J. Plant Nutr. Soil Sci. 22, 4897–4912. doi: 10.1007/s42729-022-00969-8

[B29] Muhammad I. Yang L. Ahmad S. Mosaad I. S. M. Al-Ghamdi A. A. Abbasi A. M.. (2022b). Melatonin application alleviates stress-induced photosynthetic inhibition and oxidative damage by regulating antioxidant defense system of maize: a meta-analysis. Antioxidants. 11, 512. doi: 10.3390/antiox11030512, PMID: 35326162 PMC8944576

[B30] Muhammad I. Yang L. Ahmad S. Farooq S. Khan A. Muhammad N.. (2023). Melatonin-priming enhances maize seedling drought tolerance by regulating the antioxidant defense system. Plant Physiol. 191 (4), 2301–2315., PMID: 36660817 10.1093/plphys/kiad027 PMC10069899

[B31] Nelissen H. Sun X. H. Rymen B. Jikumaru Y. Kojima M. Takebayashi Y.. (2018). The reduction in maize leaf growth under mild drought affects the transition between cell division and cell expansion and cannot be restored by elevated gibberellic acid levels. Plant Biotechnol. J. 16, 615–627. doi: 10.1111/pbi.12801, PMID: 28730636 PMC5787831

[B32] Ng C. Wang Z. Leung A. Ni J. (2019). A study on effects of leaf and root characteristics on plant root water uptake. Géotechnique. 69 (2), 151–157.

[B33] Opoku V. A. Yawson D. O. Asare P. A. Afutu E. Kotochi M. C. Amoah K. K.. (2022). Root hair and rhizosheath traits contribute to genetic variation and phosphorus use efficiency in cowpea (*Vigna unguiculata* L. Walp). Rhizosphere. 21, 100463. doi: 10.1016/j.rhisph.2021.100463

[B34] Ortega J. K. E. (2010). Plant cell growth in tissue. Physiol. Plantarum. 154, 1244–1253. doi: 10.1104/pp.110.162644, PMID: 20739609 PMC2971603

[B35] Osakabe Y. Kajita S. Osakabe K. (2011). Genetic engineering of woody plants: Current and future targets in a stressful environment. Physiol. Plantarum. 142, 105–117. doi: 10.1111/j.1399-3054.2011.01451.x, PMID: 21288247

[B36] Qi W.-Z. Liu H.-H. Liu P. Dong S.-T. Zhao B.-Q. So H. B.. (2012). Morphological and physiological characteristics of corn (Zea mays L.) roots from cultivars with different yield potentials. Eur. J. Agron. 38, 54–63.

[B37] Roelfsema M. R. Hedrich R. Geiger D. (2012). Anion channels: Master switches of stress responses. Trends Plant Sci. 17, 221–229. doi: 10.1016/j.tplants.2012.01.009, PMID: 22381565

[B38] Schlüter U. Mascher M. Colmsee C. Scholz U. Bräutigam A. Fahnenstich H.. (2012). Maize source leaf adaptation to nitrogen deficiency affects not only nitrogen and carbon metabolism but also control of phosphate homeostasis. Plant Physiol. 160, 1384–1406. doi: 10.1104/pp.112.204420, PMID: 22972706 PMC3490595

[B39] Schneider H. M. Postma J. A. Kochs J. Daniel P. Lynch J. P. Van D. D. (2020). Spatio-temporal variation in water uptake in seminal and nodal root systems of barley plants grown in soil. Front. Plant Sci. 11, 1–13. doi: 10.3389/fpls.2020.01247, PMID: 32903494 PMC7438553

[B40] Sharma M. Pang J. Wen Z. De-Borda A. Kim H. S. Liu Y.. (2021). A significant increase in rhizosheath carboxylates and greater specific root length in response to terminal drought is associated with greater relative phosphorus acquisition in chickpea. Plant Soil. 460, 51–68. doi: 10.1007/s11104-020-04776-x

[B41] Shi J. Wu X. Wang X. Zhang M. Han L. Zhang W.. (2020). Determining threshold values for root-soil water weighted plant water deficit index based smart irrigation. Agric. Water Manage. 230, 105979. doi: 10.1016/j.agwat.2019.105979

[B42] Su W. Liu B. Liu X. W. Li X. K. Ren T. Cong R. H.. (2015). Effect of depth of fertilizer banded-placement on growth, nutrient uptake and yield of oilseed rape (*Brassica napus* L.). Euro. J. Agron. 62, 38–45. doi: 10.1016/j.eja.2014.09.002

[B43] Sun G. Dong Z. Li G. Yuan H. Liu J. Yao X.. (2023). Mn3o4 nanoparticles alleviate ROS-inhibited root apex mitosis activities to improve maize drought tolerance. Adv. Biol. 7 (7), 2200317. doi: 10.1002/adbi.202200317, PMID: 36949542

[B44] Voothuluru P. Mäkelä P. Zhu J. M. Yamaguchi M. Cho I.-J. Oliver M. J.. (2020). Apoplastic hydrogen peroxide in the growth zone of the maize primary root. increased levels differentially modulate root elongation under well-watered and water-stressed conditions. Front. Plant Sci. 11. doi: 10.3389/fpls.2020.00392, PMID: 32373139 PMC7186474

[B45] Waltz E. (2014). Beating the heat. Nat. Biotechnol. 32, 610–613. doi: 10.1038/nbt.2948, PMID: 25004222

[B46] Wang F. B. Liu J. C. Zhou L. J. Pan G. Li Z. W. Zaidi S. H. R.. (2016). Senescence-specific change in ROS scavenging enzyme activities and regulation of various SOD isozymes to ROS levels in psf mutant rice leaves. Plant Physiol. Bioch. 109, 248–261. doi: 10.1016/j.plaphy.2016.10.005, PMID: 27756006

[B47] Wang J. Wen X. F. Zhang X. Y. Li S. G. Zhang D. Y. (2018). Co-regulation of photosynthetic capacity by nitrogen, phosphorus and magnesium in a subtropical Karst Forest in China. Sci. Rer-UK. 8, 7406. doi: 10.1038/s41598-018-25839-1, PMID: 29743619 PMC5943327

[B48] Wang Y. Zhang X. Y. Chen J. Chen A. J. Wang L. Y. Guo X. Y.. (2019). Reducing basal nitrogen rate to improve maize seedling growth, water and nitrogen use efficiencies under drought stress by optimizing root morphology and distribution. Agric. Water Manage. 212, 328–337. doi: 10.1016/j.agwat.2018.09.010

[B49] Wen Z. Pang J. Ryan M. H. Shen J. Siddique K. H. Lambers H. (2021). In addition to foliar manganese concentration, both iron and zinc provide proxies for rhizosheath carboxylates in chickpea under low phosphorus supply. Plant Soil. 465, 31–46. doi: 10.1007/s11104-021-04988-9

[B50] Wu P. Liu F. Li H. Wang J. Y. Liu Y. H. Gao Y.. (2022). Suitable fertilization depth can improve the water productivity and maize yield by regulating development of the root system. Agric. Water Manage. 271, 107784. doi: 10.1016/j.agwat.2022.107784

[B51] Xia Z. Q. Si L. Y. Jin Y. Fu Y. F. Wang Q. Lu H. D. (2021a). Effects of root zone temperature increase on physiological indexes and photosynthesis of different genotype maize seedlings. Russ. J. Plant Physiol. 68, 169–178. doi: 10.1134/S1021443721010180

[B52] Xia Z. Q. Zhang G. X. Zhang S. B. Wang Q. Fu Y. F. Lu H. D. (2021b). Efficacy of root zone temperature increase in root and shoot development and hormone changes in different maize genotypes. Agriculture-Basel. 11, 477. doi: 10.3390/agriculture11060477

[B53] Xia H. Y. Zhao J. H. Sun J. H. Bao G. X. Christie P. Zhang F. S.. (2013). Dynamics of root length and distribution and shoot biomass of maize as affected by intercropping with different companion crops and phosphorus application rates. Field Crops Res. 150, 52–62. doi: 10.1016/j.fcr.2013.05.027

[B54] Yamaguchi-Shinozaki K. Shinozaki K. (2006). Transcriptional regulatory networks in cellular responses and tolerance to dehydration and cold stresses. Annu. Rev. Plant Biol. 57(1), 781–803. doi: 10.1146/annurev.arplant.57.032905.105444, PMID: 16669782

[B55] York L. M. Nord E. A. Lynch J. P. (2013). Integration of root phenes for soil resource acquisition Integration of root phenes for soil resource acquisition. Front. Plant Sci. 4, 355. doi: 10.3389/fpls.2013.00355, PMID: 24062755 PMC3771073

[B56] Zhang W. Li S. Q. Shen Y. F. Yue S. C. (2021). Film mulching affects root growth and function in dryland maize-soybean intercropping. Field Crops Res. 271, 108240. doi: 10.1016/j.fcr.2021.108240

[B57] Zhang G. Meng W. Pan W. Han J. Liao Y. (2021). Effect of soil water content changes caused by ridge-furrow plastic film mulching on the root distribution and water use pattern of spring maize in the Loess Plateau. Agric. Water Manage. 241, 107338. doi: 10.1016/j.agwat.2021.107338

