# Supplementary material for: Optimizing water and nitrogen management improves maize productivity by regulating root development in the cold semi-arid Songnen plains of Northeast China
Source: Front Plant Sci. 2025 Sep 19;16:1658353. doi: 10.3389/fpls.2025.1658353 (PMC12491213; doi:10.3389/fpls.2025.1658353)
Supplement: Supplementary file 1 [file DataSheet1.docx]

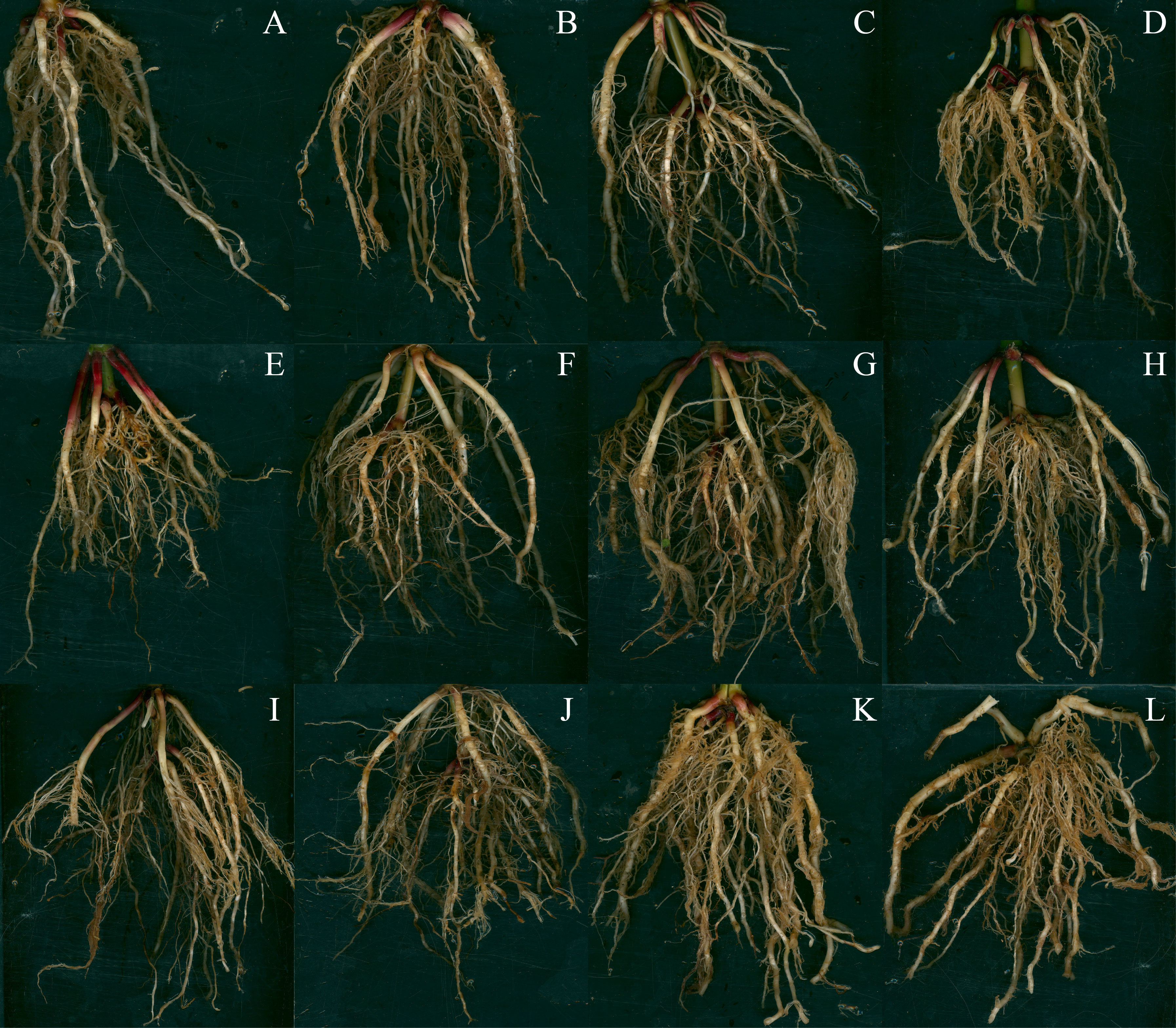


**FIGURE S1**

Effects of different water conditions and nitrogen application on maize root growth. (**A-D**) Root growth at N0, N100, N200, and N300 levels under S1 water conditions. (**E-H)** Root growth at N0, N100, N200, and N300 levels under S2 water conditions. (**I-L)** Root growth at N0, N100, N200, and N300 levels under S3 water conditions.

Note: S1 maintained the soil moisture at approximately 40% of the field holding capacity (FHC); S2 and S3 indicated that the soil moisture was maintained at approximately 60% and 80% of the FHC, respectively. N0: having no N; N100, N200, and N300 indicate having 100 kg N ha^-1^, 200 kg N ha^-1^ and 300 kg N ha^-1^, respectively.**Table S1 Effects of water-nitrogen interaction on root growth of maize in 2020 and 2021.**

| Year (Y) | Water (W) | Nitrogen (N) | RL (cm) | RLD (mm·cm-^3^) | R/S ratio | SSA (cm^2^·cm^3^) | RD (mm·plant^-1^) | No.RT (plant^-1^) |
| --- | --- | --- | --- | --- | --- | --- | --- | --- |
|  |  |  |  |  |  |  |  |  |
| 2020 | S1 | N0 | 224.93ef | 0.1176f | 0.0746f | 0.0552g | 0.2455f | 1746.67g |
|  |  | N100 | 413.41cd | 0.2161c | 0.0981e | 0.0804ef | 0.4628cd | 2787.00d |
|  |  | N200 | 422.61c | 0.2209c | 0.1003de | 0.1099e | 0.4877cd | 2996.00d |
|  |  | N300 | 459.38c | 0.2535bc | 0.1088de | 0.1190e | 0.5034c | 3319.33c |
|  | S2 | N0 | 260.45e | 0.1462e | 0.0797ef | 0.0675fg | 0.3281e | 2005.33f |
|  |  | N100 | 503.32bc | 0.2631bc | 0.1171d | 0.1303d | 0.5149bc | 3344.33c |
|  |  | N200 | 580.17b | 0.3033b | 0.14289bc | 0.1754bc | 0.5629b | 3842.67b |
|  |  | N300 | 515.62bc | 0.2696bc | 0.1368c | 0.1673c | 0.5425bc | 3680.00bc |
|  | S3 | N0 | 347.28d | 0.1816d | 0.0892e | 0.0787f | 0.4021d | 2316.00e |
|  |  | N100 | 507.86bc | 0.2655bc | 0.1304c | 0.1532cd | 0.539bc | 3494.00c |
|  |  | N200 | 747.25a | 0.3939a | 0.1795a | 0.2043a | 0.6016a | 4552.33a |
|  |  | N300 | 621.50ab | 0.3249b | 0.1564b | 0.1839b | 0.5838ab | 4305.67ab |
| 2021 | S1 | N0 | 206.21f | 0.1078g | 0.0507g | 0.0382h | 0.2864d | 1162.67h |
|  |  | N100 | 338.54de | 0.1829e | 0.0732e | 0.0705f | 0.5412bc | 2142.00e |
|  |  | N200 | 360.10d | 0.1883e | 0.0856d | 0.0884e | 0.5661bc | 2467.33de |
|  |  | N300 | 413.19cd | 0.2216d | 0.0929d | 0.1038de | 0.5939b | 2614.00d |
|  | S2 | N0 | 237.00e | 0.1239f | 0.0561fg | 0.0515g | 0.3619cd | 1391.67g |
|  |  | N100 | 423.96cd | 0.2160d | 0.1005cd | 0.1160d | 0.6053b | 2709.33cd |
|  |  | N200 | 528.74bc | 0.2764c | 0.1241bc | 0.1519b | 0.6309ab | 3384.33bc |
|  |  | N300 | 468.43c | 0.2487cd | 0.1181c | 0.1462bc | 0.6219ab | 2984.67c |
|  | S3 | N0 | 328.17de | 0.1719e | 0.0627f | 0.0589g | 0.4713c | 1756.67f |
|  |  | N100 | 442.47c | 0.2449cd | 0.1068cd | 0.1289c | 0.6123b | 2843.00c |
|  |  | N200 | 717.78a | 0.3752a | 0.1504a | 0.1975a | 0.7206a | 4364.67a |
|  |  | N300 | 582.14b | 0.3043b | 0.1371b | 0.1598b | 0.6466ab | 3654.00b |
| ANOVA | |  |  |  |  |  |  |  |
| Y | |  | * | NS | * | * | * | * |
| W | |  | *** | *** | *** | *** | *** | *** |
| N | |  | *** | *** | *** | *** | *** | *** |
| Y×W | |  | NS | NS | NS | NS | NS | NS |
| Y×N | |  | * | NS | NS | NS | * | NS |
| W×N | |  | ** | NS | * | ** | * | * |
| Y×W×N | |  | NS | NS | NS | NS | NS | NS |

Note: Root length (RL), root length density (RLD), root/shoot ratio (R/S), root specific surface area (RSSA), root diameter (RD), and number of root tips (RT) at different water-nitrogen interactions at the maturity stage during 2020 and 2021. S1 maintained the soil moisture at approximately 40% of the field holding capacity (FHC); S2 and S3 indicated that the soil moisture was maintained at approximately 60% and 80% of the FHC, respectively. N0: having no N; N100, N200, and N300 indicate having 100 kg N ha^-1^, 200 kg N ha^-1^ and 300 kg N ha^-1^, respectively. Mean values followed by different letters are significantly different at *p* < 0.05. **p* < 0.05. ***p* < 0.01. ****p* < 0.001. NS indicates not significant.

**TABLE S2 Effects of water-nitrogen interaction on root ions in 2020 and 2021.**

| Year (Y) | Water (W) | Nitrogen (N) | Na^+^ (mg·kg^-1^ DW) | K^+^ (mg·kg^-1^ DW) | Ca^2+^ (mg·kg^-1^ DW) | Mg^2+^ (mg·kg^-1^ DW) |
| --- | --- | --- | --- | --- | --- | --- |
|  |  |  |  |  |  |  |
| 2020 | S1 | N0 | 40.25a | 1.27g | 2.39f | 1.82h |
|  |  | N100 | 33.08b | 2.96f | 4.99e | 4.99f |
|  |  | N200 | 29.39c | 4.44ef | 6.36de | 6.69ef |
|  |  | N300 | 25.21cd | 4.76ef | 7.90d | 7.90e |
|  | S2 | N0 | 38.64ab | 1.68fg | 2.99f | 2.57g |
|  |  | N100 | 23.61d | 5.94e | 8.73d | 8.73e |
|  |  | N200 | 16.10ef | 10.11bc | 13.86c | 13.86c |
|  |  | N300 | 19.88e | 9.18c | 11.38c | 11.38d |
|  | S3 | N0 | 36.62ab | 2.41f | 3.56ef | 3.57fg |
|  |  | N100 | 21.49de | 7.62d | 10.62cd | 9.62de |
|  |  | N200 | 12.52g | 13.86a | 20.38a | 20.71a |
|  |  | N300 | 15.89f | 11.59b | 16.86b | 17.19b |
| 2021 | S1 | N0 | 50.65a | 0.48g | 0.89h | 0.97h |
|  |  | N100 | 44.94b | 1.44ef | 2.87g | 3.21fg |
|  |  | N200 | 40.58bc | 2.34e | 4.57f | 4.57f |
|  |  | N300 | 37.41c | 2.99e | 5.78ef | 5.78ef |
|  | S2 | N0 | 48.78ab | 0.59fg | 1.25gh | 1.31h |
|  |  | N100 | 35.43cd | 4.17de | 6.61e | 6.61e |
|  |  | N200 | 21.13f | 8.34bc | 11.74c | 11.07c |
|  |  | N300 | 30.16e | 7.41c | 9.25cd | 9.25d |
|  | S3 | N0 | 46.037ab | 0.81f | 2.01g | 2.39g |
|  |  | N100 | 33.09d | 5.86d | 8.50d | 8.50de |
|  |  | N200 | 12.11g | 12.09a | 18.26a | 18.26a |
|  |  | N300 | 18.09fg | 9.16b | 14.74b | 15.40b |
| ANOVA | |  |  |  |  |  |
| Y | |  | * | * | * | * |
| W | |  | *** | *** | *** | *** |
| N | |  | *** | *** | *** | *** |
| Y×W | |  | NS | NS | NS | NS |
| Y×N | |  | * | NS | NS | NS |
| W×N | |  | * | *** | *** | *** |
| Y×W×N | |  | NS | NS | NS | NS |

Note: Sodium ion (Na^+^), potassium ion (K^+^), calcium ion (Ca^2+^), and magnesium ion (Mg^2+^) at different water-nitrogen interactions at the maturity stage during 2020 and 2021. S1 maintained the soil moisture at approximately 40% of the field holding capacity (FHC); S2 and S3 indicated that the soil moisture was maintained at approximately 60% and 80% of the FHC, respectively. N0: having no N; N100, N200, and N300 indicate having 100 kg N ha^-1^, 200 kg N ha^-1^ and 300 kg N ha^-1^, respectively. Mean values followed by different letters are significantly different at *p* < 0.05. **p* < 0.05. ***p* < 0.01. ****p* < 0.001. NS indicates not significant.

**TABLE S3 Effects of water-nitrogen interaction on root hormones in 2020 and 2021.**

| Year (Y) | Water (W) | Nitrogen (N) | IAA (ng·g FW) | GA (ng·g FW) | ABA (ng·g FW) | Z+ZR (ng·g FW) |
| --- | --- | --- | --- | --- | --- | --- |
|  |  |  |  |  |  |  |
| 2020 | S1 | N0 | 1.08 f | 0.30 d | 26.20 a | 0.71 e |
|  |  | N100 | 3.01 e | 0.63 d | 21.33 ab | 1.80 d |
|  |  | N200 | 3.25 de | 0.78 cd | 20.23 ab | 1.82 c |
|  |  | N300 | 4.10 d | 0.95 c | 16.48 b | 2.09 c |
|  | S2 | N0 | 1.56 f | 0.47d | 23.63 a | 1.03 e |
|  |  | N100 | 4.43 d | 1.37 bc | 14.24 bc | 2.28 c |
|  |  | N200 | 6.72 bc | 1.67 ab | 8.19 de | 2.94 a |
|  |  | N300 | 6.08 bc | 1.56 b | 10.07 d | 2.79 a |
|  | S3 | N0 | 2.33 ef | 0.56 d | 22.58 a | 1.36 de |
|  |  | N100 | 5.59 c | 1.48 b | 12.62 c | 2.42 b |
|  |  | N200 | 8.39 a | 1.88 a | 3.72 f | 3.09 a |
|  |  | N300 | 7.07 b | 1.72 a | 6.54 e | 3.02 a |
| 2021 | S1 | N0 | 1.05 e | 0.27 e | 20.61 a | 0.62 d |
|  |  | N100 | 2.78 de | 0.59 cd | 17.08 ab | 1.49 bc |
|  |  | N200 | 3.00 de | 0.67 cd | 16.19 ab | 1.50 bc |
|  |  | N300 | 3.78 d | 0.73 cd | 14.21 ab | 1.72 b |
|  | S2 | N0 | 1.37 e | 0.42 de | 18.88 a | 0.92 c |
|  |  | N100 | 4.09 cd | 0.82 c | 11.38 b | 1.88 bc |
|  |  | N200 | 6.19 b | 1.14 b | 5.12 cd | 2.20 ab |
|  |  | N300 | 5.61 bc | 1.05 bc | 7.78 c | 2.01 b |
|  | S3 | N0 | 1.83 e | 0.52 d | 17.97 ab | 1.02 c |
|  |  | N100 | 5.17 c | 0.97 bc | 9.69 bc | 1.99 b |
|  |  | N200 | 7.74 a | 1.38 a | 3.03 d | 2.93 a |
|  |  | N300 | 6.52 b | 1.24 ab | 4.59 d | 2.31 ab |
| ANOVA | |  |  |  |  |  |
| Y | |  | NS | * | * | NS |
| W | |  | *** | *** | *** | ** |
| N | |  | *** | *** | *** | *** |
| Y×W | |  | NS | NS | NS | NS |
| Y×N | |  | NS | NS | NS | NS |
| W×N | |  | *** | ** | ** | ** |
| Y×W×N | |  | NS | NS | NS | NS |

Note. Interactive effects of water stress and nitrogen interaction on different hormones in maize roots (*Maize* L. cv. Nendan19) during 2020 and 2021. S1 maintained the soil moisture at approximately 40% of the field holding capacity (FHC); S2 and S3 indicated that the soil moisture was maintained at approximately 60% and 80% of the FHC, respectively. N0: having no N; N100, N200, and N300 indicate having 100 kg N ha^-1^, 200 kg N ha^-1^ and 300 kg N ha^-1^, respectively. Mean values followed by different letters are significantly different at *p* < 0.05. **p* < 0.05. ***p* < 0.01. ****p* < 0.001. NS indicates not significant.

**TABLE S4 Effects of water-nitrogen interaction on root osmoregulation substance, ROS in 2020 and 2021.**

| Year (Y) | Water (W) | Nitrogen (N) | O^2-^ (nmol·min^-1^·g^-1^ FW) | H_2_O_2_ (μmol·g^-1^ FW) | Pro (μg·g^-1^ FW) | SS (mg·g^-1^ FW) | MDA (μmol·g^-1^ FW) | SP (μg·g^-1^ FW) |
| --- | --- | --- | --- | --- | --- | --- | --- | --- |
|  |  |  |  |  |  |  |  |  |
| 2020 | S1 | N0 | 90.04 a | 266.28 a | 123.71 f | 12.72 h | 67.37 a | 3.27 g |
|  |  | N100 | 78.60 c | 179.73 ab | 154.07 de | 20.44 g | 50.84 c | 5.95 e |
|  |  | N200 | 77.37 c | 154.91 b | 166.09 d | 32.06 f | 42.39 d | 6.83 d |
|  |  | N300 | 70.06 d | 146.76 bc | 170.68 d | 37.89 e | 35.42 e | 7.38 c |
|  | S2 | N0 | 85.61 b | 210.89 a | 131.73 e | 14.48 h | 58.09 b | 4.83 fg |
|  |  | N100 | 68.36 de | 131.29 c | 179.84 cd | 42.31 d | 29.72 f | 7.14 cd |
|  |  | N200 | 63.68 e | 115.35 d | 224.51 be | 50.39 bc | 18.43 gh | 7.87 bc |
|  |  | N300 | 64.76 e | 119.31 cd | 206.19 c | 48.57 c | 21.67 g | 7.61 c |
|  | S3 | N0 | 82.58 b | 191.96 a | 142.61 e | 17.99 gh | 55.77 bc | 5.20 f |
|  |  | N100 | 66.30 e | 125.66 c | 190.72 cd | 47.43 e | 26.73 fg | 7.51 c |
|  |  | N200 | 47.25 g | 97.97 e | 310.42 a | 58.35 a | 15.79 h | 8.98 a |
|  |  | N300 | 61.33 f | 103.60 d | 246.28 b | 53.59 b | 20.62 g | 8.12 b |
| 2021 | S1 | N0 | 83.81 a | 302.59 a | 76.40 e | 7.52 h | 73.41 a | 1.82 g |
|  |  | N100 | 73.62 c | 271.29 c | 108.48 de | 12.13 g | 61.11 c | 3.10 e |
|  |  | N200 | 70.00 cd | 236.77 cd | 118.21 d | 17.12 f | 47.24 d | 3.31 e |
|  |  | N300 | 68.12 cd | 209.92 cd | 128.52 d | 21.75 e | 58.73 cd | 5.06 b |
|  | S2 | N0 | 80.62 ab | 290.72 b | 82.70 e | 9.16 h | 67.67 ab | 2.55 fg |
|  |  | N100 | 67.53 d | 193.85 d | 137.69 cd | 23.73 de | 40.35 e | 4.07 d |
|  |  | N200 | 60.87 e | 147.70 de | 177.21 b | 41.29 a | 30.17 g | 6.28 a |
|  |  | N300 | 62.73 e | 160.89 de | 165.18 bc | 29.73 c | 32.54 f | 5.32 b |
|  | S3 | N0 | 77.50 b | 283.05 b | 90.15 e | 11.13 gh | 64.37 b | 2.72 f |
|  |  | N100 | 65.73 d | 191.34 d | 152.58 c | 25.86 d | 36.94 ef | 4.29 c |
|  |  | N200 | 52.99 g | 108.98 e | 229.89 a | 43.77 a | 29.54 g | 6.50 a |
|  |  | N300 | 58.53 f | 135.59 e | 188.09 b | 34.49 b | 30.79 g | 5.93 ab |
| ANOVA | |  |  |  |  |  |  |  |
| Y | |  | * | ** | * | * | * | ** |
| W | |  | *** | *** | *** | *** | *** | *** |
| N | |  | *** | *** | *** | *** | *** | *** |
| Y×W | |  | NS | NS | * | * | NS | NS |
| Y×N | |  | NS | NS | NS | * | * | * |
| W×N | |  | NS | NS | * | ** | *** | *** |
| Y×W×N | |  | NS | NS | * | * | * | * |

Note: Interactive effects of water stress and nitrogen on the maize of roots (*Maize* L. cv. Nendan19) superoxide radical (O^2-^), hydrogen peroxide (H_2_O_2_), proline (Pro), soluble sugar (SS), malonaldehyde (MDA), soluble protein (SP) during the 2020 and 2021. S1 maintained the soil moisture at approximately 40% of the field holding capacity (FHC); S2 and S3 indicated that the soil moisture was maintained at approximately 60% and 80% of the FHC, respectively. N0: having no N; N100, N200, and N300 indicate having 100 kg N ha^-1^, 200 kg N ha^-1^ and 300 kg N ha^-1^, respectively. Mean values followed by different letters are significantly different at *p* < 0.05. **p* < 0.05. ***p* < 0.01. ****p* < 0.001. NS indicates not significant.

**TABLE S5 Effects of water-nitrogen interaction on root antioxidant enzyme activities in 2020 and 2021.**

| Year (Y) | Wate (W) | Nitrogen(N) | SOD (U·min-1·mg FW) | POD (U·min-1·mg FW) | CAT (U·min-1·mg FW) | APX (U·min-1·mg FW) | GPX (U·min-1·mg FW) | GR (U·min-1·mg FW) |
| --- | --- | --- | --- | --- | --- | --- | --- | --- |
|  |  |  |  |  |  |  |  |  |
| 2020 | S1 | N0 | 30.05 | 12.73 | 45.14 | 21.98 | 8.25 | 11.53 |
|  |  | N100 | 45.80 | 19.28 | 81.02 | 40.02 | 11.56 | 23.24 |
|  |  | N200 | 47.41 | 23.21 | 88.46 | 43.26 | 13.69 | 24.90 |
|  |  | N300 | 49.76 | 24.18 | 90.27 | 50.01 | 20.31 | 33.17 |
|  | S2 | N0 | 34.15 | 13.61 | 54.67 | 25.52 | 9.12 | 16.60 |
|  |  | N100 | 68.51 | 25.72 | 92.38 | 51.74 | 30.02 | 33.35 |
|  |  | N200 | 80.21 | 30.05 | 111.63 | 56.59 | 37.41 | 44.69 |
|  |  | N300 | 74.27 | 27.94 | 101.68 | 53.92 | 33.96 | 42.22 |
|  | S3 | N0 | 37.11 | 16.31 | 67.29 | 31.80 | 10.58 | 20.36 |
|  |  | N100 | 69.90 | 26.10 | 95.03 | 53.15 | 32.57 | 38.83 |
|  |  | N200 | 147.92 | 36.39 | 143.19 | 81.95 | 48.01 | 69.88 |
|  |  | N300 | 114.24 | 33.66 | 130.86 | 69.21 | 44.34 | 56.85 |
| 2021 | S1 | N0 | 13.93 | 9.47 | 17.10 | 14.65 | 4.38 | 7.82 |
|  |  | N100 | 38.70 | 16.69 | 51.90 | 27.83 | 7.19 | 17.50 |
|  |  | N200 | 42.18 | 18.95 | 60.97 | 29.05 | 8.24 | 20.44 |
|  |  | N300 | 47.85 | 22.86 | 65.16 | 31.22 | 9.73 | 22.89 |
|  | S2 | N0 | 29.53 | 11.83 | 26.30 | 18.46 | 5.68 | 8.39 |
|  |  | N100 | 50.90 | 23.83 | 68.95 | 36.18 | 12.78 | 27.21 |
|  |  | N200 | 79.95 | 28.46 | 83.38 | 44.03 | 30.81 | 39.09 |
|  |  | N300 | 72.50 | 25.83 | 79.29 | 42.90 | 23.75 | 37.62 |
|  | S3 | N0 | 37.64 | 13.15 | 34.86 | 20.96 | 6.55 | 10.19 |
|  |  | N100 | 62.86 | 24.33 | 76.70 | 39.29 | 19.19 | 31.82 |
|  |  | N200 | 123.57 | 35.19 | 110.18 | 61.20 | 40.71 | 54.99 |
|  |  | N300 | 99.67 | 31.91 | 102.96 | 50.47 | 34.54 | 45.01 |
| ANOVA | |  |  |  |  |  |  |  |
| Y | |  | * | * | ** | * | * | * |
| W | |  | *** | *** | *** | *** | *** | *** |
| N | |  | *** | *** | *** | *** | *** | *** |
| Y×W | |  | NS | NS | NS | NS | NS | *** |
| Y×N | |  | NS | NS | NS | * | * | NS |
| W×N | |  | *** | *** | *** | *** | *** | *** |
| Y×W×N | |  | NS | NS | NS | NS | NS | NS |

Note: Interactive effects of water stress and nitrogen on the roots of maize (*Maize* L. cv. Nendan19) superoxide dismutase (SOD), peroxidase (POD), catalase (CAT), ascorbate peroxidase (APX), glutathione peroxidase (GPX), glutathione reductase (GR) during 2020 and 2021. S1 maintained the soil moisture at approximately 40% of the field holding capacity (FHC); S2 and S3 indicated that the soil moisture was maintained at approximately 60% and 80% of the FHC, respectively. N0: having no N; N100, N200, and N300 indicate having 100 kg N ha^-1^, 200 kg N ha^-1^ and 300 kg N ha^-1^, respectively. Mean values followed by different letters are significantly different at *p* < 0.05. **p* < 0.05. ***p* < 0.01. ****p* < 0.001. NS indicates not significant.

**TABLE S6 Effects of water-nitrogen interaction on root non-enzyme antioxidants in 2020 and 2021.**

| Year (Y) | Water (W) | Nitrogen (N) | GSSG+GSH | GSH/GSSG | ASA+DSH | ASA/DSH |
| --- | --- | --- | --- | --- | --- | --- |
|  |  |  |  |  |  |  |
| 2020 | S1 | N0 | 50.91 i | 0.28 g | 5.00 g | 0.22 f |
|  |  | N100 | 112.91 g | 0.40 f | 9.27 f | 0.32 d |
|  |  | N200 | 133.61 fg | 0.49 e | 10.34 ef | 0.39 cd |
|  |  | N300 | 150.94 f | 0.52 de | 12.18 e | 0.41 c |
|  | S2 | N0 | 70.48 h | 0.31 g | 6.54 g | 0.26 e |
|  |  | N100 | 181.11 e | 0.60 d | 15.46 d | 0.47 bc |
|  |  | N200 | 237.64 c | 0.73 c | 22.51 b | 0.54 ab |
|  |  | N300 | 216.51 d | 0.66 d | 19.28 c | 0.52 b |
|  | S3 | N0 | 88.79 h | 0.35 fg | 8.01 f | 0.29 de |
|  |  | N100 | 199.11 d | 0.63 d | 17.68 cd | 0.49 bc |
|  |  | N200 | 302.81 a | 0.96 a | 31.12 a | 0.58 a |
|  |  | N300 | 266.69 b | 0.86 b | 24.54 b | 0.56 ab |
| 2021 | S1 | N0 | 52.56 h | 0.23 f | 3.02 h | 0.15 f |
|  |  | N100 | 76.99 f | 0.34 e | 6.00 f | 0.25 de |
|  |  | N200 | 89.64 f | 0.44 de | 7.43 e | 0.29 d |
|  |  | N300 | 102.18 ef | 0.47 d | 8.47 de | 0.31 d |
|  | S2 | N0 | 64.74 g | 0.29 f | 4.16 g | 0.20 e |
|  |  | N100 | 124.00 e | 0.50 d | 10.03 d | 0.35 cd |
|  |  | N200 | 189.36 bc | 0.71 c | 17.53 bc | 0.49 b |
|  |  | N300 | 170.64 c | 0.66 cd | 14.54 c | 0.41 c |
|  | S3 | N0 | 68.18 g | 0.30 f | 5.06 f | 0.20 e |
|  |  | N100 | 152.06 d | 0.58 e | 12.82 cd | 0.39 c |
|  |  | N200 | 263.88 a | 0.91 a | 25.52 a | 0.57 a |
|  |  | N300 | 217.08 b | 0.80 b | 20.76 b | 0.53 ab |
| ANOVA | |  |  |  |  |  |
| Y | |  | * | * | * | * |
| W | |  | *** | *** | *** | *** |
| N | |  | *** | *** | *** | *** |
| Y×W | |  | NS | NS | NS | NS |
| Y×N | |  | *** | NS | NS | ** |
| W×N | |  | *** | *** | *** | *** |
| Y×W×N | |  | ** | NS | NS | NS |

Note: Interactive effects of water stress and N on total glutathione, GSH/GSSG ratio, total ascorbic acid, and ASA/DHA ratio content of roots in 2020 and 2021. S1 maintained the soil moisture at approximately 40% of the field holding capacity (FHC); S2 and S3 indicated that the soil moisture was maintained at approximately 60% and 80% of the FHC, respectively. N0: having no N; N100, N200, and N300 indicate having 100 kg N ha^-1^, 200 kg N ha^-1^ and 300 kg N ha^-1^, respectively. Mean values followed by different letters are significantly different at *p* < 0.05. **p* < 0.05. ***p* < 0.01. ****p* < 0.001. NS indicates not significant.
